# A Two‐Stage Cascading Amplification Strategy Based on Zn^2+^‐Doped WO_X_ Nanozymes for Ultrasensitive Lateral Flow Immunoassays of Clostridium Difficile Toxin B

**DOI:** 10.1002/advs.202519130

**Published:** 2025-10-30

**Authors:** Junhua Su, Zhenzhen Liu, Renjie Xiong, Renlong zhu, Zijin Tong, Yufei Liu, Rui Xiao

**Affiliations:** ^1^ State Key Laboratory of Pathogen and Biosecurity Academy of Military Medical Sciences Beijing 100071 China; ^2^ Key Laboratory of Optoelectronic Technology & Systems (Chongqing University) Ministry of Education Chongqing 400044 China; ^3^ Center for Intelligent Sensing Technology College of Optoelectronic Engineering Chongqing University Chongqing 400044 China

**Keywords:** bioassay, electronic structure, ion doping, lateral flow immunoassays, peroxidase mimic, WO_X_

## Abstract

*Clostridium difficile* is one of the primary causative agents of nosocomial antibiotic‐associated diseases. Early detection and prevention are effective strategies to curb disease transmission, which often requires highly sensitive point‐of‐care testing (POCT) methods. Although lateral flow immunoassay (LFIA) technology—known for its convenience, speed, and cost‐effectiveness—has gained prominence in POCT, its application in early screening remains limited due to relatively low sensitivity. Herein, a two‐stage cascading enhancement strategy based on reductase‐like and peroxidase‐like activities of nanozyme to construct a colorimetrically enhanced LFIA platform is proposed. Specifically, Zn^2+^‐doped WO_X_ (Zn/WO_X_) loaded with Au nanoparticles (Zn/WO_X_@Au) first generates an initial colorimetric signal through immunochromatography. Subsequently, in situ light‐induced deposition of Pt creates Zn/WO_X_@Au@Pt, which exhibits enhanced peroxidase‐like activity based on optimal band structure between the components for colorimetric signal amplification. As a result, a 500‐fold enhancement in the visual limit of detection (LOD) is achieved. Moreover, the fitted LOD based on grayscale analysis for *Clostridium difficile* toxin B (Tcd B) reaches 0.01 ng mL^−1^. This strategy not only maintains the operational simplicity of LFIA but also considerably strengthens the colorimetric signal, offering a novel approach for highly sensitive colorimetric‐enhanced LFIA detection.

## Graphical abstract

Based on the modulation of active sites and electronic structure of substrate materials, along with a colorimetric two‐stage cascading enhancement strategy, an LFIA platform with highly efficient POD‐mimetic enzymes is developed, achieving highly sensitive detection of *Clostridium difficile* toxin B.

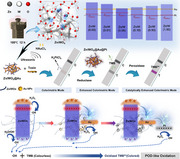

## 1 Introduction

*Clostridium difficile* toxin B (Tcd B) disrupts intestinal lining cells, leading to inflammation, cell death, and a range of clinical symptoms. It poses a serious threat to individuals on antibiotics or with compromised immune systems. Efficient detection methods are crucial for achieving early diagnosis and prevention. Existing techniques typically include cell culture,^[^ ^1^ ^]^ polymerase chain reaction (PCR),^[^ ^2^ ^]^ enzyme‐linked immunosorbent assay (ELISA),^[^ ^3^ ^]^ and LFIA.^[^ ^4^, ^5^, ^6^ ^]^ Cell culture and PCR often require complex, time‐consuming pre‐processing procedures and expensive equipment.^[^ ^7^ ^]^ In comparison, ELISA requires less time and less expensive instrumentation, but its incubation time remains unsuitable for POCT applications.^[^ ^8^ ^]^ Developing POCT for Tcd B is essential for public health and critical care management.^[^ ^9^ ^]^ LFIA offers a convenient, rapid, and economical platform well‐suited for POCT. However, its limited sensitivity often leads to false‐negative results in low‐concentration samples.^[^ ^10^ ^]^ To enhance the sensitivity of LFIA, studies have explored using nanozymes with peroxidase (POD)‐like activity as nanotags for antigen detection, combined with chromogenic substrates to intensify colorimetric signals and improve the detection limit. This approach is termed the Catalytically Enhanced Colorimetric Mode.^[^ ^11^, ^12^ ^]^

The sensitivity of colorimetric signals closely depends on the activity of nanozyme nanotags. Noble metals (e.g., Au, Pt)^[^ ^13^ ^]^ and metal oxides (e.g., Fe_3_O_4_, Co_3_O_4_)^[^ ^14^ ^]^ have been widely investigated as core materials for POD‐mimicking enzymes. Studies using noble metal nanozymes alone in immunochromatography are limited, as nanomaterials with high activity tend to aggregate, reducing active sites. Even initially stable dispersions are easily disrupted by biomolecules. Recent research focuses on using well‐dispersed nanomaterials as substrates to support noble metal nanoparticles, thereby exposing highly active surfaces and promoting the adsorption and reaction of substrate molecules. Examples include loading Au/Pt nanoparticles on MXene,^[^ ^15^ ^]^ Pt on Fe_3_O_4,_^[^ ^16^ ^]^ or Au on MOF.^[^ ^17^ ^]^ Using metal compounds with intrinsic nanozyme activity as carriers for noble metal nanocatalysts is an optimal strategy for enhancing performance. However, attaching noble metal nanoparticles via a mediator layer can block active sites on the substrate, prevent direct contact between the substrate and metals, and weaken synergistic catalytic effects mediated by interfacial charge transfer.^[^ ^18^ ^]^ Therefore, in situ growth methods, where noble metal nanoparticles replace some active sites on the substrate to form more efficient catalytic centers, are preferable for enhancing catalytic performance.

Further improving catalytic performance requires structural design of the substrate material. For metal oxides, oxygen vacancies are key tunable active sites that facilitate the adsorption and dissociation of H_2_O_2_ or O_2_, and promote the reductive deposition of noble metal nanoparticles.^[^ ^19^, ^20^ ^]^ Thus, a primary goal is to engineer more oxygen vacancies in the substrate. Effective methods include thermal treatment in an oxygen‐deficient atmosphere,^[^ ^21^ ^]^ chemical reduction using reducing agents,^[^ ^22^ ^]^ doping with low‐valent cations or anions to generate oxygen vacancies via charge compensation and induce the changes of electronic structure,^[^ ^23^ ^]^ plasma treatment for physical or chemophysical vacancy formation,^[^ ^24^ ^]^ photoinduction,^[^ ^25^ ^]^ and electrochemical process.^[^ ^26^ ^]^ Each approach has its own advantages, but the cost, simplicity, and stability of oxygen vacancies should be considered, so chemical reduction and low‐valent cation doping are employed in this study. Additionally, interfacial effects between the substrate and metal are also should be considered, as matched energy level structures can reduce the energy barrier for charge transfer. However, the formation of oxygen vacancies just turns out to be an effective means of regulating the electronic structure of the substrate.^[^ ^27^ ^]^

Here, non‐stoichiometric WO_X_ rich in oxygen vacancies is synthesized via Zn^2+^‐doped solvothermal reduction. And Au nanoseeds are then loaded using an ultrasound‐assisted reduction method to obtain Zn/WO_X_@Au with reductase‐like activity. Subsequently, ultrafine Pt nanoclusters are deposited in situ on the Au seeds via light‐induced reduction, resulting in highly efficient POD‐mimicking nanozymes denoted as Zn/WO_X_@Au@Pt. First, the solvothermally reduced WO_X_ inherently possesses numerous oxygen vacancies. Zn^2+^ doping further increases oxygen vacancies to maintain charge balance, meeting the demand for abundant active sites and narrowing the bandgap of WO_X_. The doping also localizes the electronic density of defect states formed by low‐valent W^5+^ ions, reduces the gap between defect states and band edges, and enhances inter‐valence charge transfer.^[^ ^28^ ^]^ Second, compared to direct loading of Pt on Zn/WO_X_, pre‐grown Au seeds can optimize lattice mismatching between Pt and Zn/WO_X_, stabilize Pt on the surface of Au, and act as charge‐transfer intermediaries, mitigating the large work function difference between Pt and Zn/WO_X_, thereby improving charge transfer efficiency. Moreover, the LSPR effect of Au and the narrow bandgap of Zn/WO_X_ can promote efficient Pt deposition under visible light. Finally, modifying Au with Pt can significantly improve the substrate affinity and catalytic efficiency of the POD‐mimicking enzyme. Under optimal electronic structure conditions, the Zn/WO_X_─Au─Pt heterostructure facilitates the directional transfer of thermal equilibrium and non‐equilibrium carriers from Zn/WO_X_ to Au and then to Pt. Defect states adjacent to the edge of the conduction band and the narrow bandgap of Zn/WO_X_ also promote the generation of thermally generated carriers under ambient conditions, which is conducive to the accumulation of more charge carriers to the pt. And charge accumulation on Pt optimizes its d‐band center, further enhancing catalytic activity.^[^ ^29^, ^30^ ^]^ Experimental characterization has confirmed that Zn/WO_X_@Au@Pt, based on optimized Zn^2+^‐doped Zn/WO_X_ exhibits stronger POD‐like catalytic efficiency than others, and the POD‐like catalytic efficiency of Zn/WO_X_@Au@Pt is stronger than Zn/WO_X_@Au and Zn/WO_X_. And simulations based on density functional theory (DFT) further demonstrated that Zn/WO_X_@Au@Pt has a lower energy barrier in the rate‐determining step of H_2_O_2_ homolysis.

When integrating the high‐performance Zn/WO_X_@Au@Pt nanotags with LFIA technology, a two‐stage cascading colorimetric enhancement strategy is adopted: the Zn/WO_X_@Au with reductase‐like activity is first utilized for initial colorimetric signal generation, followed by in situ deposition of Pt nanoparticles via a photo‐induced reduction process. The smaller‐sized nanotags are beneficial to the flow performance of the LFIA, while the deposition of Pt itself can also enhance the colorimetric signal. Compared to the colorimetric signal generated by Zn/WO_X_@Au alone (LOD = 50 ng mL^−1^), the light‐induced formation of Zn/WO_X_@Au@Pt results in a fivefold improvement in the visual limit of detection, achieving a visual LOD of 10 ng mL^−1^. Subsequent enzymatic chromogenic development catalyzed by the POD‐like activity of Zn/WO_X_@Au@Pt further improves the visual LOD to 0.1 ng mL^−1^, representing an additional 100‐fold increase in visual sensitivity. The Zn/WO_X_@Au@Pt‐based LFIA platform establishes a rapid and highly sensitive detection system for *Clostridium difficile* toxin B (Tcd B). This strategy of optimizing nanozyme performance through regulating active sites and the electronic structure of the substrate material to enhance the property of catalytic colorimetric detection offers a new perspective for related research.

## 2 Results and Discussion

### 2.1 Electronic Structure Regulation of WO_X_ through Zn^2+^ Doping

**Figure 1:**
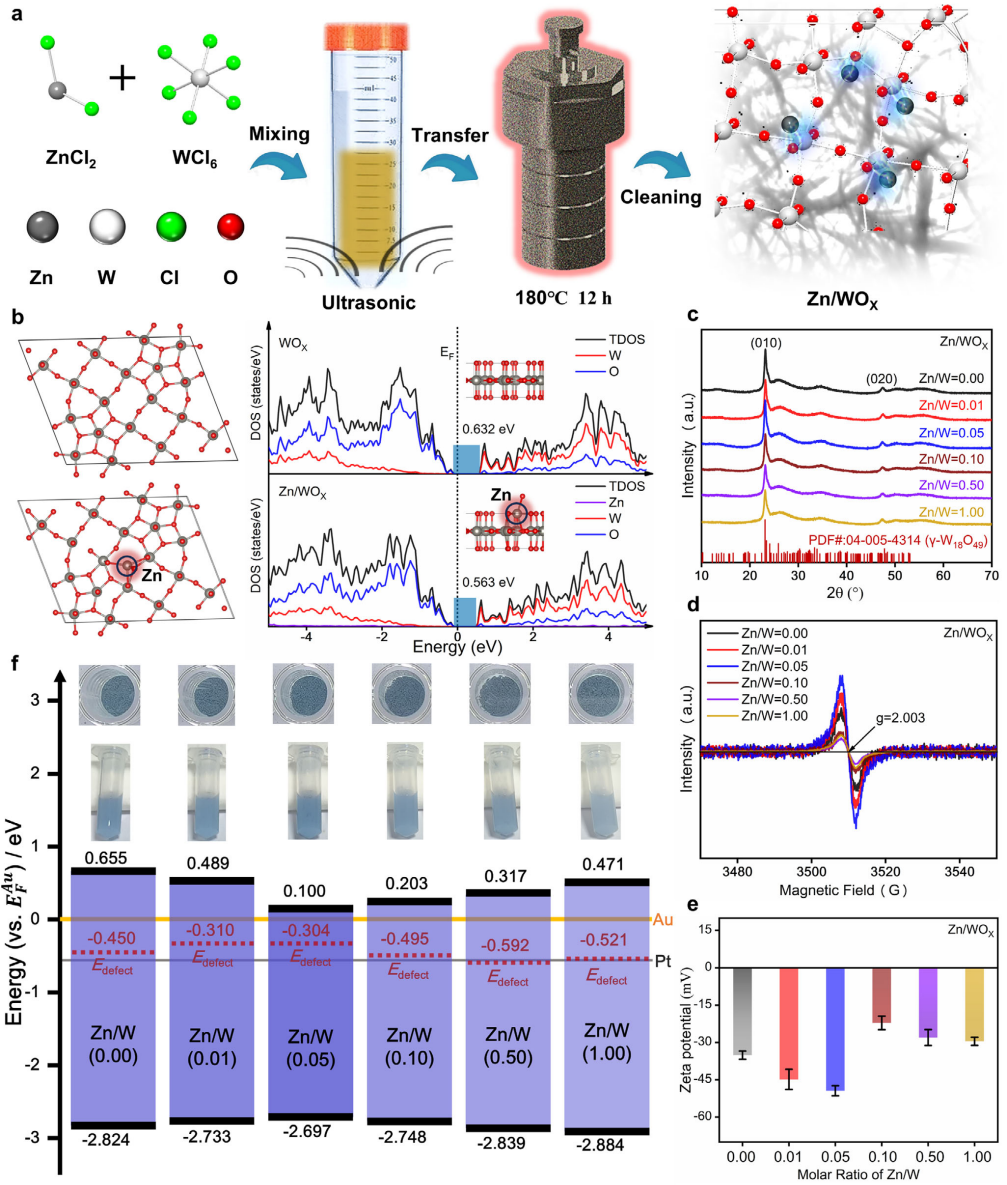
Electronic band structure modulation of WO_X_. a) Schematic diagram of the synthesis process for Zn^2+^‐doped WO_X_. b) Atomic models and calculated density of states of pure WO_X_ and Zn/WO_X_. c) XRD patterns, d) EPR spectra, e) Zeta potential values of WO_X_ with different Zn^2+^ doping concentrations. f) Optical photographs (upper panel) and schematic band structure diagrams (lower panel) of WO_X_ with different Zn^2+^ doping concentrations. The Fermi levels of Au and Pt are indicated by golden and gray lines, respectively.

During the solvothermal synthesis of WO_X_, ZnCl_2_ is introduced into the precursor solution in a specific amount and reacted together to obtain Zn^2+^‐doped WO_X_ (as shown in **Figure** **1**a). The products are named based on the molar ratio of Zn to W elements used, where, for example, Zn/W = 0 represents the sample without Zn^2+^ addition. To reveal the effects and outcomes of varying Zn^2+^ doping levels in WO_X_, various material characterization techniques and simulation calculations are carried out.

The X‐ray diffraction (XRD) pattern shown in Figure 1c confirms the crystal structure of the Zn/WO_X_ products, which primarily corresponds to the monoclinic γ‐phase W_18_O_49_. The diffraction peaks at 23.20° and 47.90° are assigned to the (010) and (020) crystal planes, respectively.^[^ ^31^ ^]^ Given the efficient photochromic/electrochromic effects of W_18_O_49_‐based “smart window” materials, the coloration–bleaching cycle induced by light irradiation and thermal annealing also serves as an intuitive method to verify its identity, as demonstrated in Figure S1 (Supporting Information).^[^ ^32^ ^]^ No diffraction peaks corresponding to Zn^2+^ compounds are observed in the XRD pattern. However, the results of energy‐dispersive spectroscopy (EDS) (Figure S3, Supporting Information) confirm the successful incorporation of Zn^2+^, suggesting that Zn^2+^ doping did not form a secondary phase or that any secondary phase is long‐range disordered. Quantitative analysis of the Zn 2p X‐ray photoelectron spectroscopy (XPS) data (Figure S4, Supporting Information) further confirms the presence of Zn^2+^ as a trace dopant, with the atomic percentage of Zn reaching only 0.70% even at the highest zinc chloride addition. Considering the following factors: 1. The ionic radius of Zn^2+^ with coordination number (CN) of 4 is 0.74 Å, and it increases with higher coordination numbers—reaching 0.82 Å at CN = 5 and 0.88 Å at CN = 6; 2. In W_18_O_49_, W^6+^/W^5+^ primarily occupies sites with CN = 6, with ionic radii distributed between 0.65 and 0.74 Å; 3. The structure of W_18_O_49_ is inherently loose, featuring abundant void spaces and large interlayer spacing.^[^ ^33^, ^34^, ^35^ ^]^ If Zn^2+^ is to substitute W^6+^/W^5+^ sites of CN = 6, it would cause significant lattice distortion, contradicting the principle of energy minimization. Therefore, Zn^2+^ doping should primarily occur via interstitial incorporation. A magnified view of the (010) diffraction peak from Figure 1c is presented in Figure S2 (Supporting Information). With increasing Zn^2+^ doping, the (010) peak exhibits a slight shift toward lower angles, indicating an expansion in the interplanar spacing, which is consistent with interstitial doping. The largest peak shift is observed for the Zn/W = 0.05 sample. Beyond this doping level (e.g., Zn/W = 0.10), the shift abruptly decreases, possibly due to exceeding the solid solubility limit leading to the precipitation of Zn^2+^ and partial recovery of the (010) peak position. Precipitated Zn^2+^ may migrate to high‐energy sites such as grain boundaries, dislocations, or surfaces, forming nanoscale secondary phases that are undetectable due to their small size or disordered nature. Furthermore, an increase in such nanoscale secondary phases at defective sites can enhance lattice distortion, which may explain the recurrence of a low‐angle shift in the (010) peak in samples with Zn/W ≥ 0.10.

Figure 1d shows the electron paramagnetic resonance (EPR) spectra of Zn/WO_X_ with different Zn^2+^ doping levels. The signal at *g* = 2.003 corresponds to oxygen vacancy defects. Semi‐quantitative analysis indicates that the oxygen vacancy content increases with higher Zn^2+^ doping, until a sharp decrease is observed at Zn/W = 0.10, demonstrating the significant impact of Zn^2+^ doping on the oxygen vacancies in WO_X_. A similar trend is observed in the O 1s and W 4f high‐resolution XPS spectra, as shown in Figure S5 (Supporting Information). The proportion of the sub‐peak at 532.51 eV, attributed to oxygen vacancies, increases with Zn^2+^ doping until a sharp decline occurs at Zn/W = 0.10. Similarly, the proportion of sub‐peaks at 37.35 and 34.45 eV, assigned to low‐valence W^5+^, also increases with Zn^2+^ doping before dropping sharply at Zn/W = 0.10.^[^ ^36^ ^]^ These observations align with the solid solubility limit conclusion drawn from XRD analysis. To maintain charge balance, Zn^2+^ doping induces the formation of oxygen vacancies and low‐valence W^5+^. The changes observed up to Zn/W = 0.10 result from continuous Zn^2+^ doping increasing oxygen vacancy and W^5+^ content. At Zn/W = 0.10, Zn^2+^ exceeds the solid solubility limit and precipitates, leading to a sharp decrease in Zn^2+^ content within the bulk phase. This phenomenon also reduces the need for oxygen vacancies and W^5+^ to compensate charge, resulting in a sudden drop in their concentrations. The slight variation in oxygen vacancy content in samples beyond Zn/W = 0.10 may be attributed to interface defects introduced by precipitated phases.^[^ ^37^, ^38^ ^]^ Figure 1e shows the zeta potential values of Zn/WO_X_ with different Zn^2+^ doping levels. The negative potential of non‐stoichiometric WO_X_ in water is primarily caused by surface hydroxylation ionization induced by defect‐state W^5+^/W^4+^.^[^ ^39^, ^40^ ^]^ Oxygen vacancy‐rich Zn/WO_X_ containing more low‐valence W^5+^/W^4+^ further enhances surface hydroxylation ionization in aqueous systems, resulting in a more negative potential. Thus, as Zn^2+^ doping concentration increases, the zeta potential of the Zn/W = 0.05 sample reaches the most negative value. This is due to increased low‐valence W^5+^/W^4+^ from charge compensation induced by Zn^2+^ doping, which promotes surface hydroxylation ionization. This is consistent with XPS results. However, with further increase in Zn^2+^ doping concentration, the potential of the Zn/W = 0.10 sample becomes more positive. This can be attributed to two reasons based on previous analysis: 1) Precipitation of Zn^2+^ reduces the local electron density associated with low‐valence W^5+^/W^4+^, weakening the surface hydroxylation ionization. 2) Precipitated Zn^2+^ on the surface shields some high‐energy sites of WO_X_, reducing the degree of surface hydroxylation ionization. Based on the second point, the further negative shift in potential for Zn/W = 0.50 and Zn/W = 1 samples can be explained by hydroxylation ionization of precipitated Zn^2+^ related phases. This is further supported by O 1s high‐resolution XPS spectra (Figure S5a–f, Supporting Information), FTIR spectroscopy (Figure S6, Supporting Information), and Raman spectroscopy (Figure S7, Supporting Information). In Figures S5a–f (Supporting Information), the proportion of the sub‐peak at 531.31 eV, assigned to surface ─OH groups associated with low‐valence W^5+^, also increases with Zn^2+^ doping until a sharp decline at Zn/W = 0.10, followed by a renewed increase.^[^ ^41^, ^42^ ^]^ This trend is fully consistent with the changes in zeta potential. In the FTIR spectra (Figure S6, Supporting Information), two peaks at 1261 and 1095 cm^−1^ become more prominent with increasing Zn^2+^ doping. The former is attributed to surface hydroxyl vibration modes, while the latter corresponds to Zn─O─W vibration modes formed between doped Zn^2+^ and W─O^−^.^[^ ^43^ ^]^ In the bulk phase of WO_X_ with high oxygen vacancy content, it is difficult to retain a large number of W─O^−^ dangling bonds. Therefore, the Zn─O─W vibration mode should primarily be associated with precipitated Zn^2+^. For W_18_O_49_, its disordered structure contains W─O─W bonds with varying lengths, resulting in two broad, featureless Raman peaks in the ranges of 100–400 and 600–900 cm^−1^. However, under environmental oxygen, laser‐induced thermal effects promote oxidation and a gradual transition to a WO_3_ (monoclinic γ‐phase) structure, leading to the appearance of characteristic peaks. During partial oxidation, four main peaks appear at 131, 267, 714, and 803 cm^−1^, while sharp peaks at 87 and 327 cm^−1^ emerge after complete oxidation.^[^ ^44^, ^45^, ^46^ ^]^ Thus, the Raman spectra shown in Figure S7 (Supporting Information) actually correspond to oxidized WO_X_. Zn^2+^ doping affects W‐O related vibration modes, causing corresponding peak shifts. With increasing Zn^2+^ doping, the peak shifts are most significant for the Zn/W = 0.05 sample. At Zn/W = 0.10, the peak shifts noticeably decrease, and all peaks become significantly broadened or nearly disappear. The broadening and disappearance of Raman peaks in WO_X_ indicate reduced oxidation degree, suggesting that samples beyond Zn/W = 0.10 experience significantly less laser‐induced thermal oxidation. The only possible compensating shielding agent against thermal oxidation is likely the precipitated Zn^2+^‐related secondary phase.

In addition to influencing the crystal structure, oxygen vacancy content, chemical states, and molecular vibration modes of WO_X_, Zn^2+^ doping further modulates its electronic band structure. Figure 1f shows a schematic band structure diagram constructed based on UV–vis absorption spectroscopy (Figure S8, Supporting Information) and XPS valence band spectra (Figure S9, Supporting Information). It can be observed that the bandgap gradually narrows with increasing Zn^2+^ doping, reaching a minimum at Zn/W = 0.05, after which it gradually widens again. This trend aligns with changes in oxygen vacancy content induced by Zn^2+^ doping and precipitation, indicating that interstitial Zn^2+^ doping reduces the bandgap of WO_X_ by promoting oxygen vacancy formation, while subsequent Zn^2+^ precipitation diminishes this effect.^[^ ^47^ ^]^ Compared to the parent monoclinic WO_3_, W_18_O_49_ exhibits a more disordered structure. Its amorphous nature and high defect density lead to broad electronic density of states near the band edges and introduce defect energy levels, often resulting in a mixed direct and indirect bandgap character. This is reflected in the UV–vis absorption spectra (Figure S8a, Supporting Information) by a low‐slope absorption edge with tailing (purple region) and a broad absorption peak in the near‐infrared region (red region) attributed to polaron hopping between W^5+^ defect states and W^6+^ sites.^[^ ^28^ ^]^ With Zn^2+^ doping, the UV absorption edge of Zn/WO_X_ becomes smoother and steeper, reflecting band edge localization induced by Zn^2+^ doping. Combined with changes in the defect state peak positions in the valence band spectra (Figure S9, Supporting Information), it is evident that Zn^2+^ doping also affects the defect energy levels of WO_X_. This corresponds precisely to the variation in low‐valence W^5+^/W^4+^ content induced by charge compensation. Moreover, the primary defect states in W_18_O_49_ are constituted by these low‐valence W^5+^/W^4+^ species.^[^ ^32^ ^]^ This also explains why the blue color of Zn/WO_X_ deepens in the optical images in the upper part of Figure 1f. Furthermore, photoluminescence excitation and emission spectra provide additional evidence for the strong regulatory effect of Zn^2+^ on the electronic structure of WO_X_, as shown in Figure S10 (Supporting Information). In the emission spectra, the broad peak centered at 440 nm indicates widened and delocalized band edges, where excited electrons undergo diverse and complex relaxation processes before radiative recombination. The broad near‐infrared emission ≈715 nm is likely due to relaxation‐recombination emission from defect energy levels formed by low‐valence W^5+^/W^4+^, as the broad near‐infrared absorption in the UV–vis spectra suggests a wide distribution of these defect states, sufficient to form near‐infrared emission at ≈715 nm with the delocalized band edges. After Zn^2+^ doping, the intensity and range of both the 440 and 715 nm emission peaks are suppressed and reduced, indicating that Zn^2+^ promotes localization of both band‐edge and defect state electronic distributions. In the excitation spectra, the optimal excitation wavelength for band‐to‐band electron–hole recombination emission is ≈376 nm for all samples. The tailing peak below 375 nm corresponds to relaxation‐recombination emission from shallower energy levels above the conduction band edge, potentially involving synergistic continuous processes of interband and intraband excitation.^[^ ^48^ ^]^ As Zn^2+^ doping localizes the band edges, the tailing part of the excitation spectrum is significantly suppressed. Additionally, with increasing Zn^2+^ doping concentration, the optimal excitation wavelength red‐shifts, with the extent of red‐shift decreasing beyond Zn/W = 0.10, consistent with the bandgap changes derived from UV–vis absorption spectra.

From a theoretical perspective, we construct models of undoped WO_X_ and Zn^2+^‐doped WO_X_, and calculate their electronic density of states (DOS) based on first‐principles calculations (Computational method is detailed in the “Density Functional Theory (DFT) Calculations” section of the Supporting Information). As shown in Figure 1b, the labeled “Zn” represents an interstitially doped Zn^2+^ ion. The DOS diagram reveals that after Zn^2+^ doping, the original band‐edge DOS of WO_X_—dominated by O 2p and W 3d orbitals at −0.518 and 0.716 eV, respectively—becomes more localized toward the band gap, resulting in band gap narrowing. Meanwhile, DOS outside the range of −0.518 to 0.716 eV shifts deeper into the bands. These observations indicate, first, that Zn^2+^ doping strongly influences the electronic structure of the outer orbitals of WO_X_. Second, they provide solid theoretical support for the experimentally observed band edge localization induced by Zn^2+^ doping. Finally, although the DOS contribution from the 4s and 3d orbitals of the doped Zn^2+^ itself is low, it still triggers a significant reconstruction of the electronic states of WO_X_. This confirms that even trace‐level interstitial Zn^2+^ doping can effectively modulate the electronic band structure of WO_X_.

### 2.2 Mechanism of Colorimetric Two‐Stage Cascading Enhancement

For LFIA, the smooth migration of nanotags is critical. Under otherwise identical conditions, smaller‐sized nanotags are generally more conducive to capillary‐driven chromatography.^[^ ^49^, ^50^ ^]^ However, smaller nanotags also imply a reduction in active components, which can lead to a decrease in colorimetric signal intensity. From a hydrodynamic perspective, WO_X_ with a 1D nanowire morphology is capable of migrating smoothly on nitrocellulose (NC) membranes with 20 µm pores. Moreover, compared to nanoparticles, its linear structure is more easily captured by specific sites, thereby enhancing the signal‐to‐noise ratio.^[^ ^51^, ^52^ ^]^ However, after loading numerous nanoparticles onto WO_X_, this smooth migration can be hindered, making it necessary to sacrifice some signal intensity to maintain flow performance.

**Figure 2:**
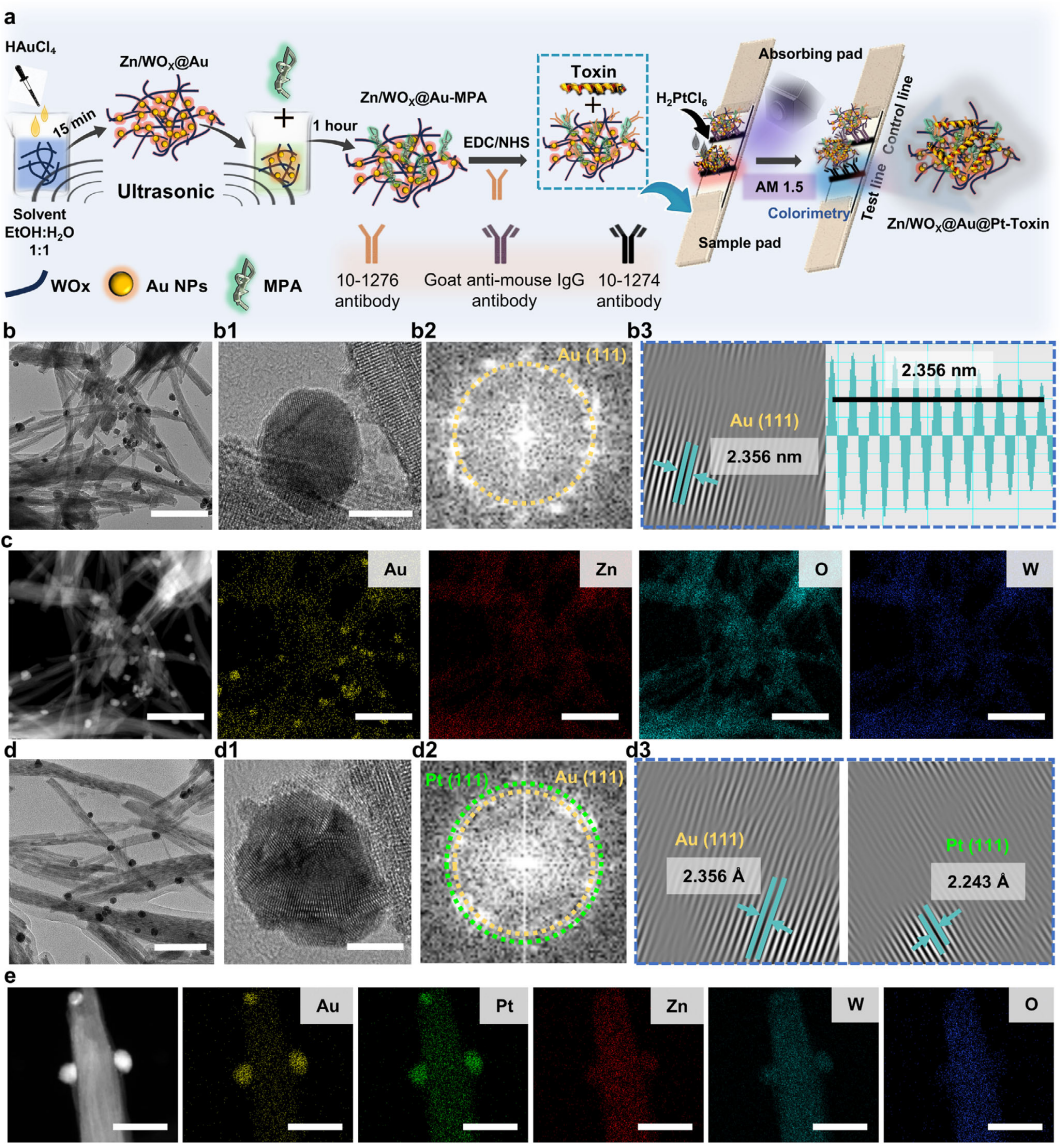
a) Schematic diagram of the colorimetric two‐stage cascading enhancement process; b) TEM image, b1) HR‐TEM image, b2) selected area electron diffraction pattern, b3) lattice fringe pattern, and c) EDS mapping of Zn‐doped WO_X_ after loading with Au seeds; d) TEM image, d1) HR‐TEM image, d2) SAED pattern, d3) lattice fringe pattern, and e) EDS mapping of Zn/WO_X_@Au after loading with Pt nanoclusters. Scale bar of (b,c) is 100 nm, Scale bar of (b1) is 10 nm, Scale bar of (d,d1) is 100 and 5 nm, Scale bar of (e) is 40 nm.

However, the colorimetric two‐stage cascading enhancement strategy effectively addresses the issues mentioned above, as illustrated in **Figure** **2**a (for detailed immunochromatographic procedures, refer to the “Operation and Detection of LFIA Sensors” section in the Supporting Information). First, Au seeds ≈10 nm in size are loaded onto Zn/WO_X_ nanowires (Zn/W = 0.05) via an ultrasound‐assisted reduction method, resulting in Zn/WO_X_@Au reductase‐mimicking nanozyme nanotags. These are subsequently functionalized to conjugate with 10‐1276 antibodies. Specifically, 3‐mercaptopropionic acid (MPA) is attached to the surface of Zn/WO_X_@Au via Au─S bonds, followed by covalent conjugation between the carboxyl groups of MPA and the amino groups of the 10‐1276 antibodies using carbodiimide crosslinking. The antibody‐conjugated Zn/WO_X_@Au (Zn/WO_X_@Au‐antibody) can specifically capture Tcd B antigen. Meanwhile, the test line (T) on the LFIA strip is also modified with 10‐1274 antibodies, enabling specific capture of Tcd B antigen. After adding a mixture of Tcd B antigen and Zn/WO_X_@Au‐antibody to the strip, a 10‐minute chromatographic process allows Zn/WO_X_@Au to be captured firmly at the T line, generating a colorimetric signal. For clarity, this mode of detection using Zn/WO_X_@Au reductase‐mimicking nanozyme nanotags is defined as the Colorimetric Mode (CM); Next, 30 µL of 0.0122 mol L^−1^ chloroplatinic acid solution is added to the strip and allowed to migrate, followed by irradiating the T zone with an AM 1.5 light source (optical power: 320 mW) for 5 min. Leveraging the reductase‐mimicking activity and photocatalytic effect of Zn/WO_X_@Au, Pt nanoclusters are rapidly grown in situ on the Au seeds, forming Zn/WO_X_@Au@Pt POD‐mimicking nanozymes. Although extended irradiation could further increase Pt loading, a 5‐minute irradiation is selected to meet POCT requirements while maintaining high catalytic activity (optimization of chloroplatinic acid volume and irradiation duration is detailed in the “Operation and Detection of LFIA Sensors” section of the Supporting Information). The deposition of Pt nanoclusters further enhances the colorimetric signal generated in CM, defining a new stage termed enhanced colorimetric mode (ECM). Finally, a prepared 3,3′,5,5′‐tetramethylbenzidine (TMB) color‐developing solution is added to the T zone already functionalized with Zn/WO_X_@Au@Pt POD‐mimicking nanozymes. The highly efficient POD‐like activity rapidly oxidizes colorless TMB into a blue product, further amplifying the colorimetric signal. This final stage is defined as the catalytically enhanced colorimetric mode (CECM). This two‐step strategy—first deploying small‐sized nanotags for migration, followed by in situ growth of fully functional nanotags at the T line—effectively avoids the flow obstruction issues common with pre‐loaded high‐content nanotags. It provides a practical pathway for applying non‐0D nanotag materials in LFIA and is highly compatible with multifunctional detection modes such as catalytically enhanced colorimetry,^[^ ^12^ ^]^ fluorescence enhancement,^[^ ^53^ ^]^ and SERS.^[^ ^54^ ^]^

To confirm the successful loading of Au onto Zn/WO_X_ and subsequent deposition of Pt onto Zn/WO_X_@Au, a series of characterization techniques is performed, including transmission electron microscopy (TEM), high‐resolution TEM (HR‐TEM), EDS, and selected‐area electron diffraction (SAED). Figure 2b shows a TEM image of Zn/WO_X_@Au after 15 minutes of ultrasound‐assisted reduction, demonstrating the successful loading of Au seeds. SAED is performed on the dark nanoparticle (Figure 2b1), and the resulting diffraction spots, shown in Figure 2b2, are indexed to the Au (111) crystal plane. Further analysis using inverse fast Fourier transform (IFFT) reveals lattice fringes corresponding to the Au (111) plane (Figure 2b3), with a measured interplanar spacing of 2.356 Å, consistent with standard values for Au (111). SAED and corresponding IFFT lattice fringe analysis are also conducted on the nanowire region (as shown in Figure S11, Supporting Information), reconfirming that the Zn/WO_X_ structure belongs to the W_18_O_49_ crystal phase. Additionally, EDS mapping presented in Figure 2c visually confirms the presence of Au on the Zn/WO_X_ support. Similarly, Zn/WO_X_@Au@Pt nanotags—synthesized by subjecting Zn/WO_X_@Au (prepared via 15‐minute ultrasound reduction) to 5 minutes of light‐induced Pt deposition—are thoroughly characterized. The TEM image in Figure 2d clearly shows nanoparticles anchored on the nanowires. A close‐up view in Figure 2d1 distinctly reveals the formation of a secondary phase consisting of Pt nanoclusters grown around the Au seeds. SAED and IFFT analysis of characteristic regions identifies diffraction spots and lattice fringes corresponding to both Au (111) and Pt (111) crystal planes (Figure 2d2,d3), confirming the successful deposition of Pt onto the Au seeds. EDS mapping in Figure 2e also visually verifies the presence of Pt on the Zn/WO_X_@Au substrate. Furthermore, by extending the light‐induced deposition time to 60 min, the heterogeneous growth of Pt nanoclusters around the Au seeds could be observed more clearly, as shown in Figure S12 (Supporting Information).

**Figure 3:**
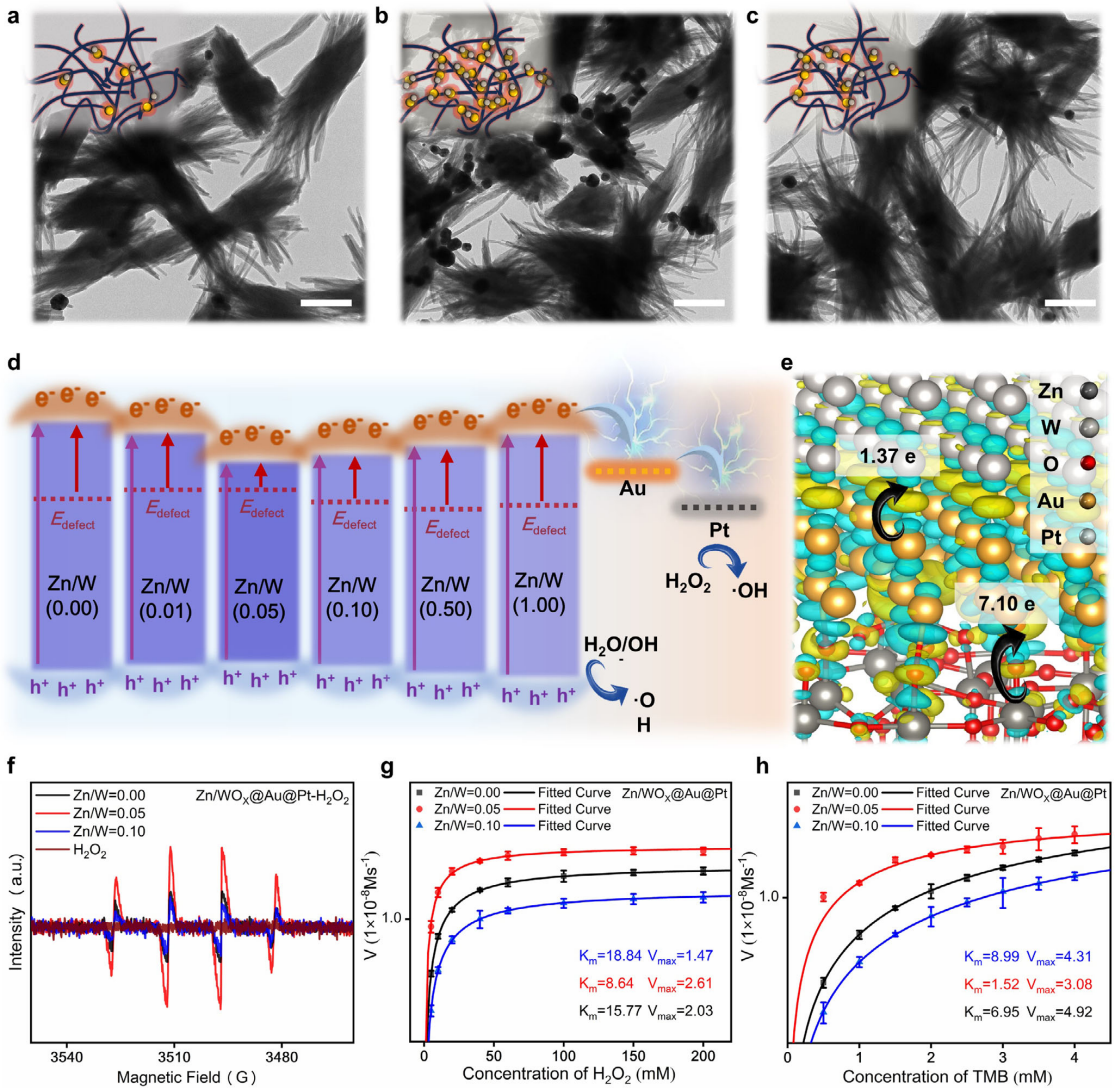
a–c) TEM images of Zn/WO_X_@Au@Pt prepared with a) Zn/W = 0, b) Zn/W = 0.05, and c) Zn/W = 0.10, Scale bar: 200 nm; d) Schematic illustration of the band structure and charge transfer in Zn/WO_X_@Au@Pt synthesized with varying Zn^2+^ doping concentrations; e) Bader charge analysis and charge density difference diagram of Zn/WO_X_@Au@Pt. Yellow regions represent charge accumulation, while blue regions indicate charge depletion; f) EPR spectra of DMPO‐·OH adducts for Zn/WO_X_@Au@Pt with Zn/W = 0, 0.05, and 0.10; Enzymatic kinetic curves of Zn/WO_X_@Au@Pt prepared with Zn/W = 0, 0.05, and 0.10: g) TMB concentration fixed at 2 mm, with H_2_O_2_ concentration increasing from 5, 10, 20, 40, 60, 100, 150 to 200 mm; h) H_2_O_2_ concentration fixed at 50 mm, with TMB concentration increasing in an arithmetic sequence starting from 0.5 mm with a common difference of 0.5 mm.

Beyond stepwise operation and in situ growth, the colorimetric two‐stage cascading enhancement strategy more importantly, enables continuous amplification of the colorimetric signal. The transition from CM to ECM and then to CECM relies fundamentally on the catalytic activity of the precursor materials. Therefore, Zn/WO_X_ (Zn/W = 0.05), which possesses the narrowest bandgap and the highest oxygen vacancy concentration, is selected as the optimal foundation and plays a critical role. First, the abundant oxygen vacancy sites and narrow bandgap of the Zn/WO_X_ (Zn/W = 0.05) sample facilitate the loading of a higher density of Au seeds within a short 15‐minute ultrasonication period, theoretically leading to a stronger colorimetric signal in CM. Second, the high density of Au seed sites on Zn/WO_X_@Au enables the subsequent deposition of a larger number of Pt nanoclusters. Moreover, the narrow bandgap of Zn/WO_X_ (Zn/W = 0.05) enhances the efficient utilization of light by the Zn/WO_X_‐Au heterostructure, promoting the generation of photoinduced charge carriers and enabling rapid nucleation and growth of Pt around the Au seeds within 5 min of irradiation. This results in a stronger ECM colorimetric signal and higher catalytic activity. As shown in **Figure** **3**a,b, TEM images of Zn/WO_X_@Au@Pt synthesized using Zn/WO_X_ (Zn/W = 0), Zn/WO_X_ (Zn/W = 0.05), and Zn/WO_X_ (Zn/W = 0.10) as substrates are labeled as a, b, and c, respectively. It can be observed that the sample based on Zn/WO_X_ (Zn/W = 0.05) achieves a higher loading of Au seeds, which in turn supports the deposition of more Pt nanoclusters. Finally, the high quantity of Zn/WO_X_@Au@Pt formed under 5‐minute light irradiation using the Zn/WO_X_ (Zn/W = 0.05) substrate ensures sufficient catalyst availability. Furthermore, the heterojunction formed in Zn/WO_X_ (Zn/W = 0.05)─Au─Pt promotes more efficient directional transfer of both thermal equilibrium and non‐equilibrium charge carriers to Pt from a kinetic perspective, thereby optimizing the catalytic efficiency of Pt toward substrate molecules (Figure 3d). DFT‐based Bader charge analysis and charge density difference calculations for Zn/WO_X_@Au@Pt further validate this charge transfer pathway. As shown in Figure 3e, ≈7.10 e is transferred from Zn/WO_X_ to Au, followed by a subsequent transfer of ≈1.37 e from Au to Pt. To experimentally validate the superior POD‐like catalytic activity of Zn/WO_X_@Au@Pt nanotags based on Zn/WO_X_ (Zn/W = 0.05), quantitative EPR tests measuring homolytic cleavage of H_2_O_2_ (Figure 3f) and enzymatic kinetic assays using H_2_O_2_ and TMB as substrates (Figure 3g,h) are conducted comparing samples prepared from Zn/WO_X_ with Zn/W ratios of 0, 0.05, and 0.10. As shown in Figure 3f, after adding equal masses of each Zn/WO_X_@Au@Pt nanotags type to identical volumes of H_2_O_2_ and reacting for 1 min, ·OH radicals are trapped and detected. The results indicate that Zn/WO_X_@Au@Pt based on Zn/WO_X_ (Zn/W = 0.05), most efficiently catalyzes the homolytic cleavage of H_2_O_2_ into ·OH, yielding the strongest EPR signal corresponding to DMPO‐·OH adducts. The enzymatic kinetic experiments with TMB and H_2_O_2_ as substrates are presented in Figure 3g,h. At low concentrations, according to the Beer–Lambert law, absorbance is linearly proportional to substrate concentration. Therefore, the optical density (OD) value at 655 nm, derived from the UV–vis spectra of oxidized TMB (Figure S15, Supporting Information), reflects changes in substrate concentration.^[^ ^55^ ^]^ The Michaelis–Menten curves in Figure 3g,h represent the production rates of oxidized TMB under varying concentrations of H_2_O_2_ and TMB, respectively. The kinetic parameters *V*_max_ and *K*_m_, extracted from the Michaelis–Menten curves, represent the maximum reaction rate (catalytic efficiency) and the Michaelis constant (substrate affinity), respectively.^[^ ^56^ ^]^ The results show that Zn/WO_X_@Au@Pt, based on Zn/WO_X_ (Zn/W = 0.05), exhibits the smallest *K*_m_ and the largest *V*_max_ for both H_2_O_2_ and TMB substrates, demonstrating the most efficient POD‐like catalytic activity. Additionally, the specific activity values of Zn/WO_X_@Au@Pt based on different Zn/W ratios, as calculated in Table S1 (Supporting Information), also demonstrate that the sample with a Zn/W ratio of 0.05 exhibits the strongest catalytic efficiency.

**Figure 4:**
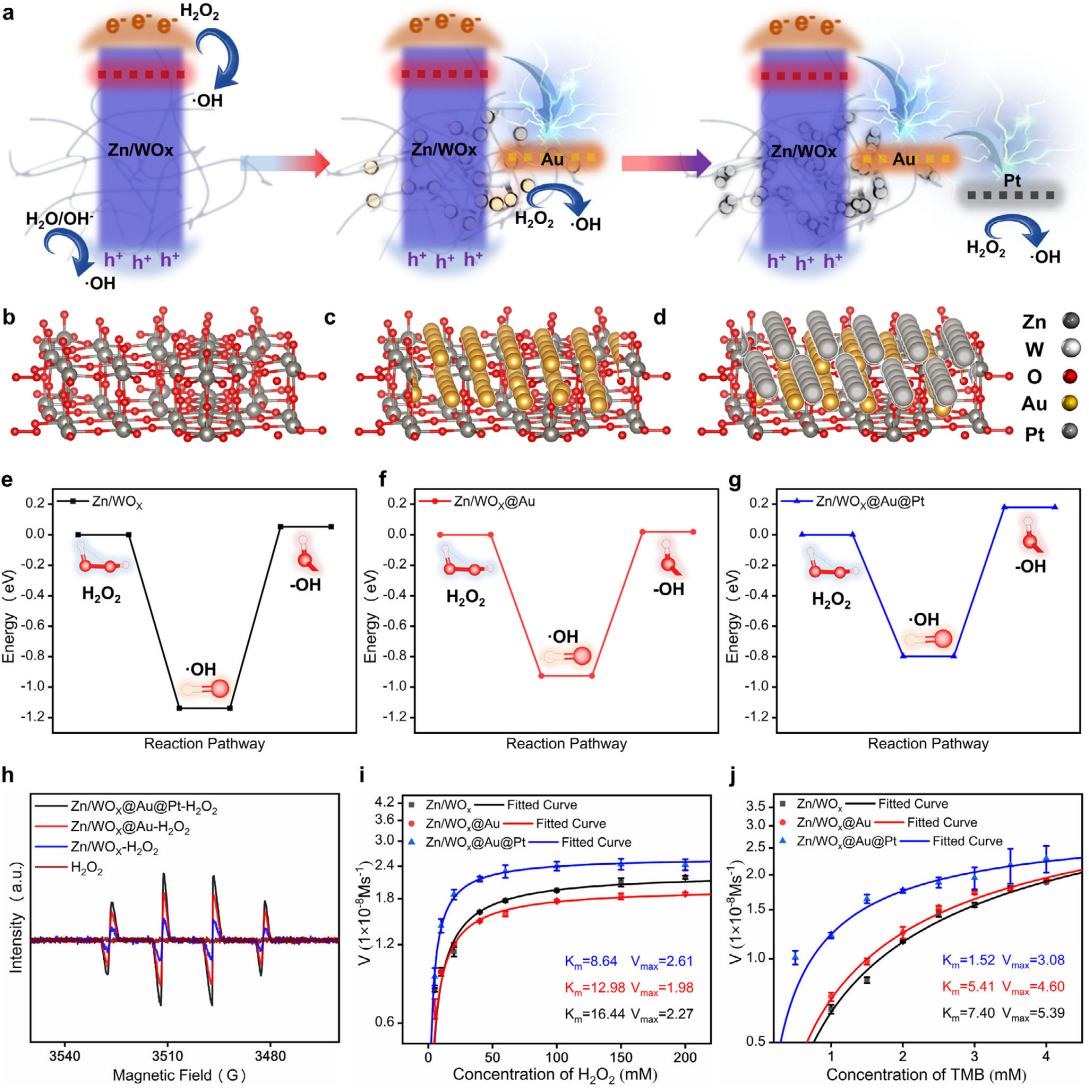
a) Schematic diagrams of the band structures and hydroxyl radical production processes of Zn/WO_X_, Zn/WO_X_@Au, and Zn/WO_X_@Au@Pt, respectively; b–d) Atomic structure models of Zn/WO_X_, Zn/WO_X_@Au, and Zn/WO_X_@Au@Pt, respectively. The exposed crystal facet of Zn/WO_X_ is (010), while those of Au and Pt are both (111); e–g) DFT‐simulated reaction pathways for the homolytic cleavage of H_2_O_2_ into ·OH catalyzed by Zn/WO_X_, Zn/WO_X_@Au, and Zn/WO_X_@Au@Pt, respectively; h) EPR spectra attributed to DMPO‐·OH adducts for Zn/WO_X_, Zn/WO_X_@Au, and Zn/WO_X_@Au@Pt, in the presence of H_2_O_2_; Enzyme kinetic curves of Zn/WO_X_, Zn/WO_X_@Au, and Zn/WO_X_@Au@Pt: i) TMB concentration fixed at 2 mm, with H_2_O_2_ concentration increasing from 5, 10, 20, 40, 60, 100, 150 to 200 mm; j) H_2_O_2_ concentration fixed at 50 mm, with TMB concentration increasing in an arithmetic sequence starting from 0.5 mm with a common difference of 0.5 mm.

As shown in **Figure** **4**a, the nanotags used in CECM are achieved through a sequential material‐building process based on CM and ECM, serving as the most critical element throughout the detection process and making the primary contribution to colorimetric signal enhancement. Here, Zn/WO_X_ and Zn/WO_X_@Au are used as control groups to further verify that Zn/WO_X_@Au@Pt exhibits stronger POD‐like activity due to the heterointerface formed in the Zn/WO_X_─Au─Pt system. First, atomic structure models of Zn/WO_X_ (Figure 4b), Zn/WO_X_@Au (Figure 4c), and Zn/WO_X_@Au@Pt (Figure 4d) are constructed from a theoretical perspective. Based on first‐principles calculations, the thermodynamic feasibility and kinetic processes of the homolytic cleavage reaction of H_2_O_2_ are evaluated for these three materials, as shown in Figure 4e–g. All three materials satisfy the thermodynamic conditions for the homolytic cleavage of H_2_O_2_, meaning the reaction can proceed spontaneously. However, Zn/WO_X_@Au@Pt exhibits a significantly lower energy barrier in the rate‐determining step of H_2_O_2_ homolysis, which kinetically favors more efficient generation of ·OH radicals. In the TMB chromogenic system based on POD activity, the rate of H_2_O_2_ homolysis into ·OH directly determines the oxidation and color development rate of TMB. Therefore, Zn/WO_X_@Au@Pt is theoretically expected to yield the strongest colorimetric signal. Subsequently, EPR spectroscopy is used to compare the ·OH generation capabilities of equal masses of Zn/WO_X_, Zn/WO_X_@Au, and Zn/WO_X_@Au@Pt when reacted with identical amounts of H_2_O_2_ for the same duration. As shown in Figure 4h, all three materials can catalyze the production of ·OH from H_2_O_2_, but the sample containing Zn/WO_X_@Au@Pt shows the strongest ·OH trapping signal, indicating the highest catalytic efficiency for ·OH generation; Finally, enzymatic kinetic assays using TMB and H_2_O_2_ as substrates are conducted to further evaluate the catalytic efficiency of the three materials, as shown in Figure 4i,j. For the H_2_O_2_ substrate, Zn/WO_X_@Au@Pt shows the smallest *K*_m_ and the largest *V*_max_, demonstrating the highest affinity and catalytic efficiency toward H_2_O_2_, and thus the most potent POD‐like activity. For the TMB substrate, Zn/WO_X_@Au@Pt exhibits the smallest *K*_m_, indicating the strongest affinity for TMB. However, its lowest *V*_max_ value suggests that, under limited H_2_O_2_ conditions, the direct oxidation efficiency for TMB of Zn/WO_X_@Au@Pt is lower compared to the other two materials. This observation aligns with the proposed mechanism in which Zn/WO_X_@Au@Pt relies on its highly efficient catalytic homolysis of H_2_O_2_ to generate ·OH, which in turn oxidizes TMB. This confirms that Zn/WO_X_@Au@Pt acts as a POD‐mimicking enzyme with high specificity and efficient catalytic function.

### 2.3 Tcd B Detection Based on Colorimetric Two‐Stage Cascading Enhancement LFIA

**Figure 5:**
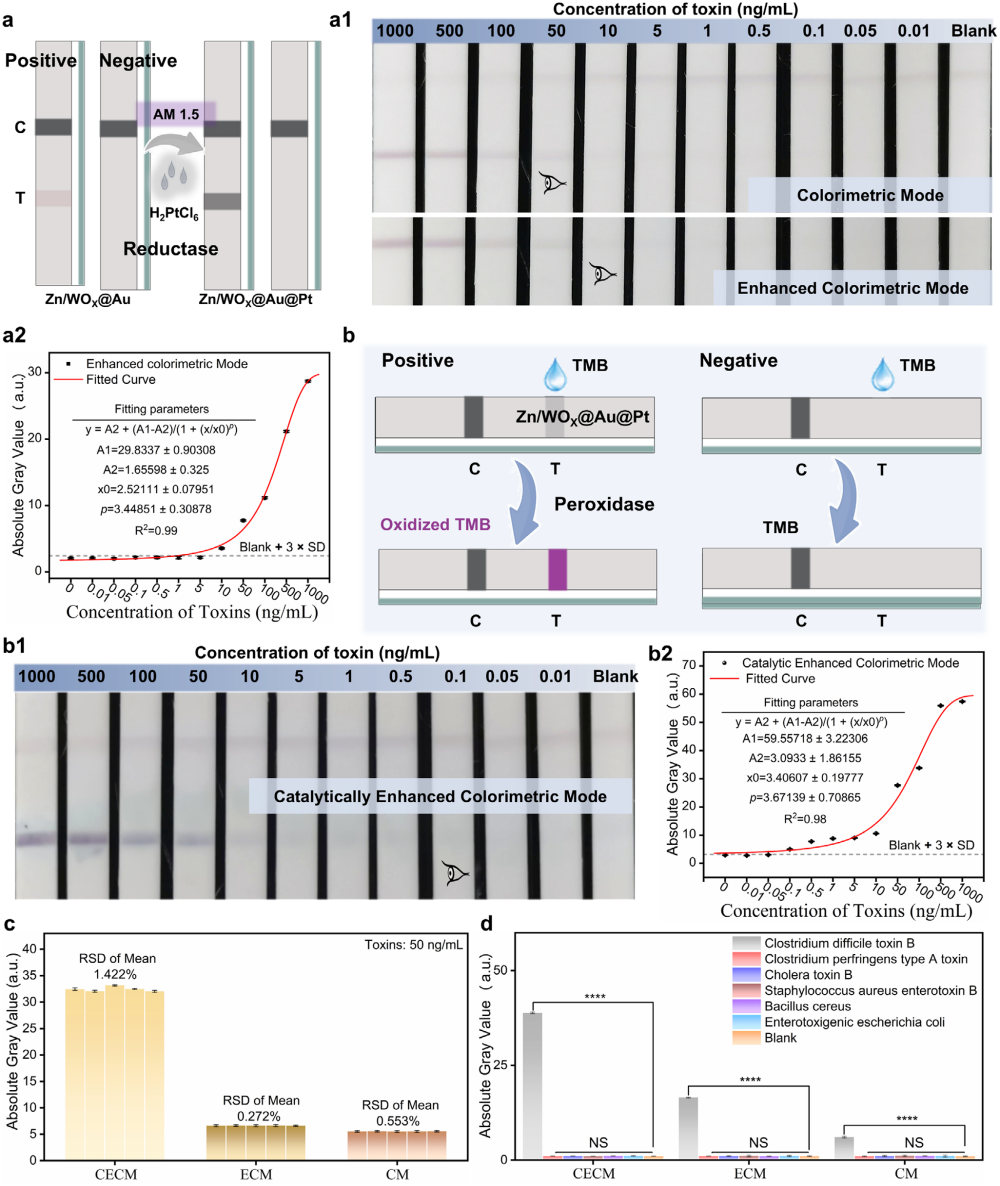
a) Schematic diagram of the operational procedure from CM to ECM: light‐induced deposition of Pt on Zn/WO_X_@Au. a1) Photographs of test strips for detecting Tcd B toxin at different concentrations under both CM and ECM modes. a2) Fitting curve of gray value versus toxin concentration derived from the gray values of the T zones extracted from test strips at different Tcd B concentrations under ECM mode (sample size *n* = 3 for gray values). b) Schematic diagram of colorimetric signal enhancement of positive samples via catalyzing the TMB color‐developing solution under CECM. b1) Photographs of test strips for detecting Tcd B toxin at different concentrations under CECM mode. b2) Fitting curve of gray value versus toxin concentration derived from the gray values of the T zones extracted from test strips at different Tcd B concentrations under CECM mode (sample size *n *= 3 for gray values). c) Repeatability validation of the three modes (CM, ECM, and CECM): five repeated detections of toxin at 50 ng mL^−1^ (sample size *n* = 3 for gray values). d) Specificity validation of the three modes (CM, ECM, and CECM): detection of seven enterotoxin‐related antigens including blank (sample size *n* = 3 for gray values), the significantly smaller *p*‐values demonstrate statistically robust selectivity for Tcd B detection: * *p *< 0.05, ** *p* < 0.01, *** *p *< 0.001, **** *p *< 0.0001. Error bars in all the pictures represent the standard deviation of three independent experiments (mean ± SD, *n* = 3), *t‐*test is used for calculating *p*‐values.

As described in Figure 2a, the colorimetric two‐stage cascading enhancement strategy is applied in LFIA (see the “Operation and Detection of LFIA Sensors” section in the Supporting Information for detailed procedures). As shown in **Figure** **5**a, Zn/WO_X_@Au‐antibody nanotags prepared using Zn/WO_X_ with Zn/W = 0.05 are mixed with the running buffer after binding with Tcd B antigen, and then added to the sample pad. After 10 min of migration, the mixture passes through both the test line and control line (C). The pre‐immobilized 10‐1274 antibody on the T captures the nanotags, producing a visible colorimetric signal. In contrast, no colorimetric signal appears when a negative sample without Tcd B antigen is tested. The detection mode resulting from this process is referred to as CM; Subsequently, 30 µL of a 0.0122 mol L^−1^ chloroplatinic acid solution dispersed in a 1:1 ethanol–water mixture is added to the sample pad and allowed to migrate for 5 min. Simultaneously, the T zone is irradiated with an AM 1.5 light source. This promotes the in situ reduction and growth of Pt nanoclusters on the Zn/WO_X_@Au reductase‐mimicking nanozyme, forming Zn/WO_X_@Au@Pt nanotags. The mode of detection after this light‐induced Pt deposition for enhanced colorimetric signal is defined as ECM. Figure 5a1 shows photographs of test strips obtained from CM and ECM detection of different concentrations of Tcd B. The visual LOD for CM is 50 ng mL^−1^, while that for ECM is 10 ng mL^−1^, representing a fivefold improvement in visual detection sensitivity. Figure 5a2 displays the grayscale values of the T zones from ECM test strips at different Tcd B concentrations and their corresponding fitting curve. Based on grayscale values, the fitted LOD reaches 5 ng mL^−1^, which indicates that theoretically, 5 ng mL^−1^ Tcd B can be detected based on the grayscale values of visual images. This is worth noting that the visual LOD is determined by naked‐eye observation and can be influenced by the set gradient of detection, whereas the grayscale value is a digital extraction of visual presentation, allowing for the theoretical LOD to be fitted based on grayscale values. Therefore, the fitted LOD based on grayscale values can be an extension of the visual LOD, indicating the highest level at which the visual LOD can theoretically be achieved.

Next, 1 µL of color‐developing solution—prepared by mixing 3 µL of 200 mm H_2_O_2_ and 10 µL of 200 mm TMB—is added to the T zone of the test strip after ECM processing. After ≈1 min of reaction, photographs are taken, and the entire 16‐minute assay is thus completed at this point. As shown in Figure 5b, if the sample is positive, TMB is oxidized and changes color due to the presence of Zn/WO_X_@Au@Pt POD‐mimicking enzyme, leading to further enhancement of the colorimetric signal. Otherwise, no change occurs. The detection mode achieved through this colorimetric signal enhancement by TMB oxidation based on POD‐mimicking enzyme is defined as CECM. Figure 5b1 displays photographs of test strips under CECM detecting different concentrations of Tcd B. The visual LOD reaches 0.1 ng mL^−1^, representing a 100‐fold improvement in sensitivity compared to ECM and a 500‐fold enhancement compared to CM. Figure 5b2 shows the grayscale values extracted from the T zones in Figure 5b1 and their corresponding fitting curve for CECM at various Tcd B concentrations. The fitted LOD based on grayscale values reaches 0.01 ng mL^−1^. Figure 5c presents the grayscale values extracted from the T zones after five repeated detections of 50 ng mL^−1^ Tcd B under CM, ECM, and CECM modes. The relative standard deviation of the five grayscale values for each mode is below 2%, indicating excellent detection reproducibility for all three modes and high consistency of the colorimetric two‐stage cascading enhancement strategy (corresponding test strip images are shown in Figure S16, Supporting Information). Figure 5d shows the grayscale values extracted from the T zones after detecting four enterotoxins (100 ng mL^−1^)—Tcd B, Clostridium perfringens type A toxin, cholera toxin B, and Staphylococcus aureus enterotoxin B—as well as two enterotoxin bacteria (10^6^ CFU mL^−1^)—Bacillus cereus and enterotoxigenic Escherichia coli—under CM, ECM, and CECM modes (corresponding test strip images are shown in Figure S17, Supporting Information). The results sufficiently demonstrate the high specificity of each detection mode and the consistency of the colorimetric two‐stage cascading enhancement strategy.

## 3 Conclusion

A colorimetric two‐stage cascading enhancement strategy based on LFIA is proposed and applied for the detection of Tcd B enterotoxin. This approach achieves a 500‐fold improvement in visual sensitivity compared to the initial colorimetric mode, with a fitted detection limit reaching 0.01 ng mL^−1^. The stepwise strategy—first using Zn/WO_X_@Au nanotags and then photo‐induced reduction to form Zn/WO_X_@Au@Pt nanotags—mitigates potential flow obstruction issues that may arise from excessive nanoparticle loading on the 1D Zn/WO_X_ nanowires during LFIA. Zn^2+^ doping induces multiple beneficial effects in WO_X_: an increase in oxygen vacancy active sites, bandgap narrowing, and localization of low‐valence W^5+^ defect states near the conduction band edge. These effects lay the foundation for high‐density Au seed loading and rapid photo‐induced Pt deposition. Moreover, the optimized kinetic conditions of Zn/WO_X_─Au─Pt heterojunction promote directional charge transfer to Pt, endowing the Zn/WO_X_@Au@Pt nanotags with enhanced POD‐like activity. Through the regulation of active sites and electronic structure in the base material, combined with the colorimetric two‐stage cascading enhancement strategy, the LFIA detection platform is expected to accommodate various morphologies of nanotags and achieve stronger colorimetric signals.

## 4 Experimental Section

Experimental details and methods are provided in the Supporting Information.

### Statistical Analysis

1) In plots with error bars (e.g., Zeta potential, gray value, etc.), all data were obtained from at least three repeated measurements (Sample size, *n* = 3). The graphs were plotted using the mean ± standard deviation (Mean ± SD) of the three replicates to represent the actual variability in the data distribution. 2) In Figure 5, the relationship between the gray value and the concentration of toxins was fitted using a logistic function with the Levenberg–Marquardt iterative algorithm. The dashed line indicated the limit of detection, calculated as the blank signal mean plus three standard deviations (Blank + 3*SD). Repeatability validation was evaluated by analyzing five independent sensor groups (Test sample size, *n* = 5) at constant Tcd B concentrations, with measurement consistency determined by calculating the relative standard deviation of response signals. Specificity validation was evaluated by testing six bacterial species and one blank item. Data underwent Shapiro–Wilk normality testing, one‐way ANOVA, followed by two‐tailed *t‐*tests versus blank controls. The significantly smaller *p‐*values demonstrated statistically robust selectivity for SA detection. 3) In Figure 3 and Figure 4, the enzyme saturation kinetic curves were fitted using the Michaelis–Menten function with the Levenberg–Marquardt iterative algorithm. 4) All statistical data processing was performed using Origin software.

## Conflict of Interest

The authors declare no conflict of interest.

## Author Contributions

J.S. and Z.L. contributed equally to this work. J.S. was mainly responsible for the experimental design and material preparation. Z.L. was mainly responsible for the pathogen detection work. R.X., R.Z., and Z.T. played a role in the final proofreading of the text and the organization of the Graphic and textual materials. Y.L. and R.X. supervised the work team and provided financial support.

## Supporting information



Supporting Information

## Data Availability

The data that support the findings of this study are available from the corresponding author upon reasonable request.
