# Supplementary material for: A Two‐Stage Cascading Amplification Strategy Based on Zn2+‐Doped WOX Nanozymes for Ultrasensitive Lateral Flow Immunoassays of Clostridium Difficile Toxin B
Source: Adv Sci (Weinh). 2025 Oct 30;13(3):e19130. doi: 10.1002/advs.202519130 (PMC12806447; doi:10.1002/advs.202519130)
Supplement: Supplementary file 1 — Supporting Information [file ADVS-13-e19130-s001.docx]

**Supporting Information**

**A Two-Stage Cascading Amplification Strategy Based on Zn^2+^-Doped WO_X_ Nanozymes for Ultrasensitive Lateral Flow Immunoassays of Clostridium difficile Toxin B**

*Junhua Su****^†^****, Zhenzhen Liu****^†^****, Renjie Xiong, Renlong zhu, Zijin Tong, Yufei Liu* and Rui Xiao**

***^†^****The authors contributed equally to this work*

ZhenZhen Liu, Renlong zhu, Zijin Tong, Rui Xiao

State Key Laboratory of Pathogen and Biosecurity, Academy of Military Medical Sciences, Beijing 100071, China

E-mail: xiaorui@bmi.ac.cn

Junhua Su, Renjie Xiong *&* Yufei Liu

Key Laboratory of Optoelectronic Technology&Systems (Chongqing University) Ministry of Education, Chongqing 400044, China

Center for Intelligent Sensing Technology, College of Optoelectronic Engineering, Chongqing University, Chongqing 400044, China

E-mail: Yufei.Liu@cqu.edu.cn

***Experiment and Method Section***

1. ***Materials and Instruments****:*

**Chemicals and Reagents**: ZnCl_2_ (Aladdin, cas:7646-85-7), WCl_6_ (Aladdin, cas:13283-01-7), Ethanol (AR), Deionized water, Tween-20 (Sigma-Aldrich), Dulbecco's Phosphate buffered saline (PBS, Sigma), Acetic acid-sodium acetate buffer solution (NaAc-HAc buffer, pH=3.6). Macroporous nitrocellulose (NC) membrane CN95 was purchased from Sartorius (Spain). Glass fiber sample pad (CB08) sample loading pad, absorbent pad and plastic backing card were supplied by Liangxin Technology Co and Shanghai Jieyi Biotechnology Co (China). Goat anti-mouse IgG (catalog#: D111024) was provided by Sangon Biotech Co., Ltd. (China). *Clostridium difficile* toxin B Monoclonal Antibody (catalog#: 10^-^1274) from Invitrogen Co. *Clostridium difficile* toxin B antibody (catalog#: 10^-^1276) from Fitzgerald Co. Chloroplatinic Acid (H_2_PtCl_6_·6H_2_O), Acetone, Hydrogen peroxide 30% aqueous solution (H_2_O_2_) from Sinopharm Chemical Reagent. 2-(N-morpholino) ethanesulfonic acid (MES), N-(3-dimethylaminopropyl)-N′-ethylcarbodiimide hydrochloride (EDC, ≥99%), 3,3′,5,5′-Tetramethylbenzidine (TMB, ≥98%), and 3-Mercaptopropionic Acid (MPA, ≥98%) were obtained from Sigma-Aldrich. Fetal bovine serum (FBS) was purchased from thermo Scientific. N-Hydroxysulfosuccinimide sodium salt (NHS, 97 %) were purchased from Alfa Aesar. Bovine serum albumin (BSA, pH 7.0) was purchased from GPC biotechnology.

**Instrumentation:** Ultraviolet-visible Absorption Spectrometer (UV-Vis, Shimadzu 2600), Nano Particle Size and Zeta Potential Analyzer (Malvern Nano-ZS90 Zetasizer), X-ray Photoelectron Spectroscopy (XPS, Thermo escalab 250Xi), X-ray Diffractometer (XRD, D8 ADVANCE X), Transmission Electron Microscope (TEM, Talos-F200s) with Energy Dispersive Spectrometer (EDS, Oxford Super X), Scanning Electron Microscope (SEM, SU8020) with Energy Dispersive Spectrometer (HORIBA EMAX mics2), Electron Paramagnetic Resonance spectra (EPR, Bruker A300^-1^0/12), Multifunctional Microplate Reader (SPARK, TECAN, Austria) was used to detect the change in the absorbance value of oxTMB at 655nm. Fourier Transform Infrared Spectrometer (FTIR, Bruker ALPHA II), Raman Spectrometer (B&W Tek, i-Raman Plus BWS465-785H spectrometer).

1. ***Synthesis of Zn/WO_X_, Zn/WO_X_@Au and Zn/WO_X_@Au@Pt:***

**2.1 Preparation of *Zn/WO_X_*:** First, 0.198 g of WCl_6_ was dissolved in 60 mL of anhydrous ethanol. A certain amount of 1 M ZnCl_2_ dispersed in anhydrous ethanol was then added to the aforementioned solution. The entire mixture was transferred to a 100 mL autoclave and reacted at 180 °C for 12 hours overnight. The resulting precipitate was collected and alternately washed twice with anhydrous ethanol and deionized water, followed by air-drying in the dark to obtain Zn/WO_X_. Pure WO_X_ was synthesized using the same method without the addition of ZnCl_2_ dispersion.

**2.2 Preparation of *Zn/WO_X_@Au*:** Next, 8 mg of the as-synthesized Zn/WO_X_ was added to 30 mL of a mixed solvent (ethanol:water = 1:1) and dispersed by ultrasonication for 15 minutes. Then, 400 μL of chloroauric acid was added, and the mixture was further ultrasonicated for 15 minutes to allow Au seeds to load onto Zn/WO_X_. The product was washed twice by alternate centrifugation with anhydrous ethanol and deionized water, yielding Zn/WO_X_@Au.

**2.3 Preparation of *Zn/WO_X_@Au@Pt*:** The Zn/WO_X_@Au precipitate from the previous step was redispersed in 30 mL of the mixed solvent (ethanol:water = 1:1) and ultrasonicated. Then, 200 μL of chloroplatinic acid solution (1 g/100 mL) was added and mixed thoroughly. Finally, under irradiation with an AM 1.5 light source (320 mW), Pt nanoclusters were reduced and loaded onto the Au seeds, resulting in the formation of Zn/WO_X_@Au@Pt.

1. ***Synthesis of Zn/WO_X_@Au-MPA***

10 mmol/L MPA ethanol solution is prepared and take 2 μL of it is added into the 400 μL Zn/WO_X_@Au ethanol solution obtained above. Then, the solution is sonicated for 1 h to promote the formation of Au-S bonds between MPA and Au. After that, the products are centrifuged and washed twice with ethanol. Finally, the Zn/WO_X_@Au-MPA precipitate is resuspend in 1 mL ethanol for later use.

1. ***Antibody modification on Zn/WO_X_@Au-MPA***

1 mL Zn/WO_X_@Au-MPA ethanol dispersion solution obtained above is centrifuged and washed once by 0.05% PBST (Volume of PBS: Tween-20 = 0.05:99.95), and then the supernatant should be removed. Take 500 μL MES (10 mmol, pH = 5.5), 50 μL EDC (10 mmol) and 100 μL NHS (10 mmol) to add into the above sample. And 15 minutes ultrasound process is need to activate the carboxyl groups on MPA. Then the supernatant is removed, and 300 μL 0.05% PBST and 3 μL antibody (10^-^1276) are added in. The solution should be sonicated for disperse. And then it is placed on a shaker (25 ℃, 800 rpm) for 2.5-3 hours. Then 100 μL 10% BSA (Volume of BSA: H_2_O = 10:90) is added and the solution is vortex mixed well, and another 1.5 hours shaking is needed for non-specific blocking. Finally, the products are washed once with 0.05% PBST, resuspend it in 200 μL 0.05% PBST and stored at 4 ℃ for future use.

1. ***Operation and Detection of LFIA Sensors***

The LFIA sensor is assembled from several parts including macroporous nitrocellulose membrane (NC), glass fiber sample pad, absorbent pad, and plastic backing card, as shown in the following Figure S18. The plastic backing card provides support function, the sample pad is used for dripping liquid samples, the NC membrane provides capillary force to push the solution system to flow on the film, and the absorbent pad can absorb excess liquid to prevent reflux. Firstly, the 10-1274 and IGg antibodies are wrote down on the NC membrane as the Test and Control lines, respectively. The 10-1274 antibody only captures Tcd B, while the IGg antibody captures almost all antigens. The signal changes of the Test line reflect the detection of the target antigen, while the Control line can reflect whether the sensor is malfunctioning; Then the NC membrane with antibodies written on is dried at 37 ℃ and the assembly of each part of the sensor is completed after drying; Afterwards, appropriate amounts of tags, antigens of different concentrations and running buffer are mixed, and then 70 μL of it are added dropwise onto the sample pad. Under capillary action, tags carrying antigens pass through the Test line and Control line and are captured. The concentration of antigens determines the amounts of tags retained on the T-line, thereby achieving quantitative detection of Tcd B; Finally, we can obtain detection results through naked eye and Grayscale value extraction. For CM, nanotags is Zn/WO_X_@Au and 10 minutes is need for the entire process. For ECM, nanotags is Zn/WO_X_@Au@Pt and 5 minutes is need for light induced reduction of 30 μL 0.0122 mol/L chloroplatinic acid dispersed in a mixed solvent of ethanol : water = 1:1. For CECM, nanotags is Zn/WO_X_@Au@Pt and 1 minutes is need for the color change of color-developing solution. It is worth noting that the antibody concentration, running buffer, and tag addition amount written on the Test line need to be optimized in advance to achieve the optimal signal-to-noise ratio. In our detection system, the antibody concentration on test line is 1.2 mg/mL, the concentration of tags is 40 mg/mL and addition volume are 1.3 μL, and the running buffer is 1% PBST (Volume of PBS: Tween-20 = 99:1), 10% BSA (Volume of BSA: H_2_O = 1:9) and FBS in a volume ratio of 9:1:0 (as shown in Figures S15d-f). And in the CECM, the selection of the light irradiation duration and the amount of chloroplatinic acid solution added was also specifically optimized. As shown in Figure S13, the loading of Pt nanoparticles increases with prolonged light induction time. UV-Vis testing of Zn/WO_X_@Au@Pt obtained under different light induction times, after catalyzing TMB for the same duration in the same system, revealed that the difference in catalytic effect between the nanotags obtained at 5 minutes and those obtained at 60 minutes was less than 10% (calculated based on the peak intensity ratio at 655 nm). Therefore, the 5-minute condition not only meets the requirements for catalytic efficiency but also satisfies the needs of rapid testing. Figure S14 shows the detection of 50 ng/mL toxin and negative controls performed under ECM and CECM conditions using test strips with different amounts of chloroplatinic acid added under 5-minute light induction. It can be observed that the condition with 30 μL of chloroplatinic acid added yields the highest signal-to-noise ratio in both modes. Thus, the amount of chloroplatinic acid added in the experiments was determined to be 30 μL.

**
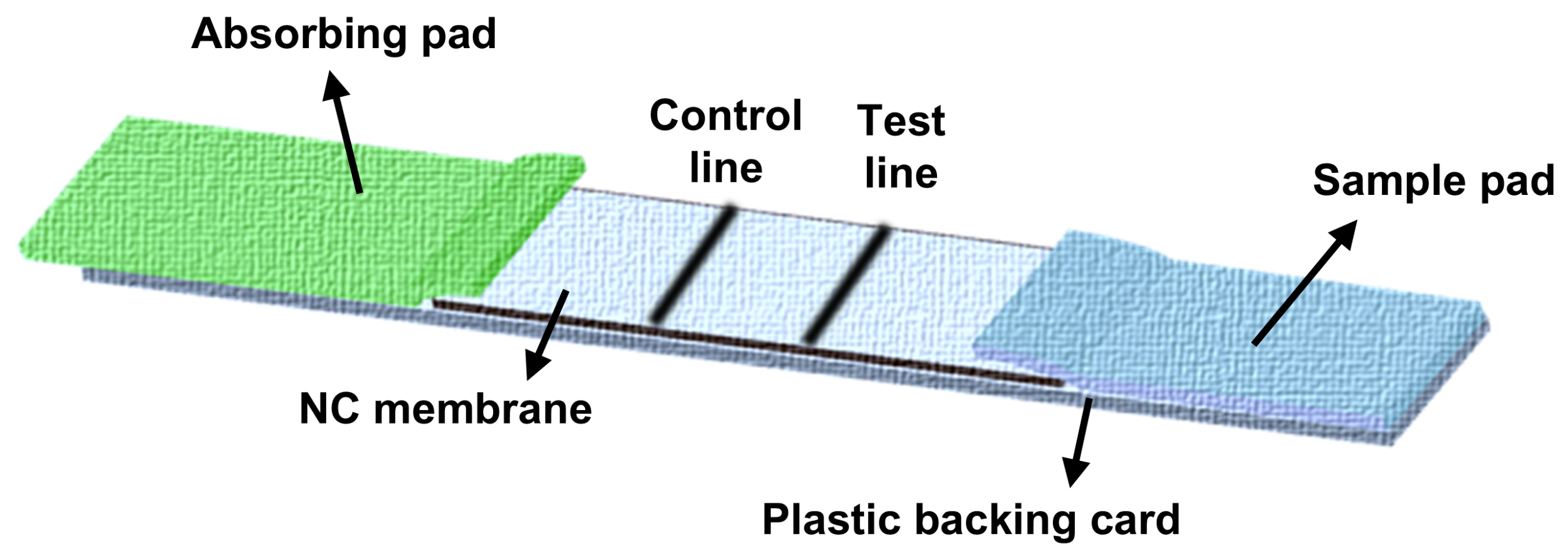
**

**Figure S18.** Schematic diagram of the compositions of LFIA sensor

1. ***Configuration of TMB colorimetric enhancement system***

For the CECM, the color-developing solution is prepared from original solutions of 200 mM TMB acetone solution, 2M H_2_O_2_ and 0.2 M NaAc-HAc buffer solution (pH=3.6). Firstly, we investigate the effects of pH value of the color development solution and the amounts of tags added on the colorimetric system, as shown in Figures S15a-c. The optimal pH value for the reaction should be around 4.8, and the tags concentration is not saturated within the range of 50 μg/mL. Therefore, the color-developing solution we chose was prepared as 3 μL 200 mM H_2_O_2_ + 10 μL 200 mM TMB + 187 μL NaAc-HAc. And then, 1 μL color development solution is added onto the T line, react for 1 minute, and take a photo to obtain the colorimetric signal. In the catalytic kinetics experiments, the total volume of the solution in the 96 well plate is 200 μL, and the solution is composed of 0.2 μL tags, 5 μL H_2_O_2_, 10 μL TMB acetone solution and 185 μL NaAc-HAc buffer solution. H_2_O_2_ and TMB solutions with different concentrations need to be prepared in advance and stored at 4 ℃. The fixed reaction system is 200 μL, ensuring the addition of 185 μL NaAc-HAc buffer to maintain stable pH, and the concentration of tags is maintained at 40 μg/mL. After preliminary experiments, the H_2_O_2_ concentration is fixed at 50 mM, a Michaelis Menten curve is obtained by increasing the TMB concentration in an arithmetic sequence with the first term of 0.5 mM and a tolerance of 0.5; and then the TMB concentration is fixed at 2 mM, another Michaelis Menten curve is obtained by increasing the H_2_O_2_ concentration from 5 mM, 10 mM, 20 mM, 40 mM, 60 mM, 100 mM, 150 mM to 200 mM. Finally, the kinetic parameters of the Michaelis Menten curve are extracted to analyze the reaction kinetics process.

1. ***Density Functional Theory (DFT) Calculations***

All calculations in this study were performed with the Vienna ab initio Simulation Package (VASP) ^[1]^ within the frame of density functional theory (DFT). The exchange-correlation interactions of electron were described via the generalized gradient approximation (GGA) with PBE functional, ^[2]^ and the projector augmented wave (PAW) method ^[3]^ was used to describe the interactions of electron and ion. Additionally, the DFT-D3 method ^[4, 5]^ was used to account for the long-range van der Waals forces present within the system. The Monkhorst-Pack scheme ^[6]^ was used for the integration in the irreducible Brillouin zone. The kinetic energy cut-off of 450 eV was chosen for the plane wave expansion. The lattice parameters and ionic position were fully relaxed, and the total energy was converged within 10^-5^ eV per formula unit. The final forces on all ions are less than 0.02/Å.

***Supplementary Figures***


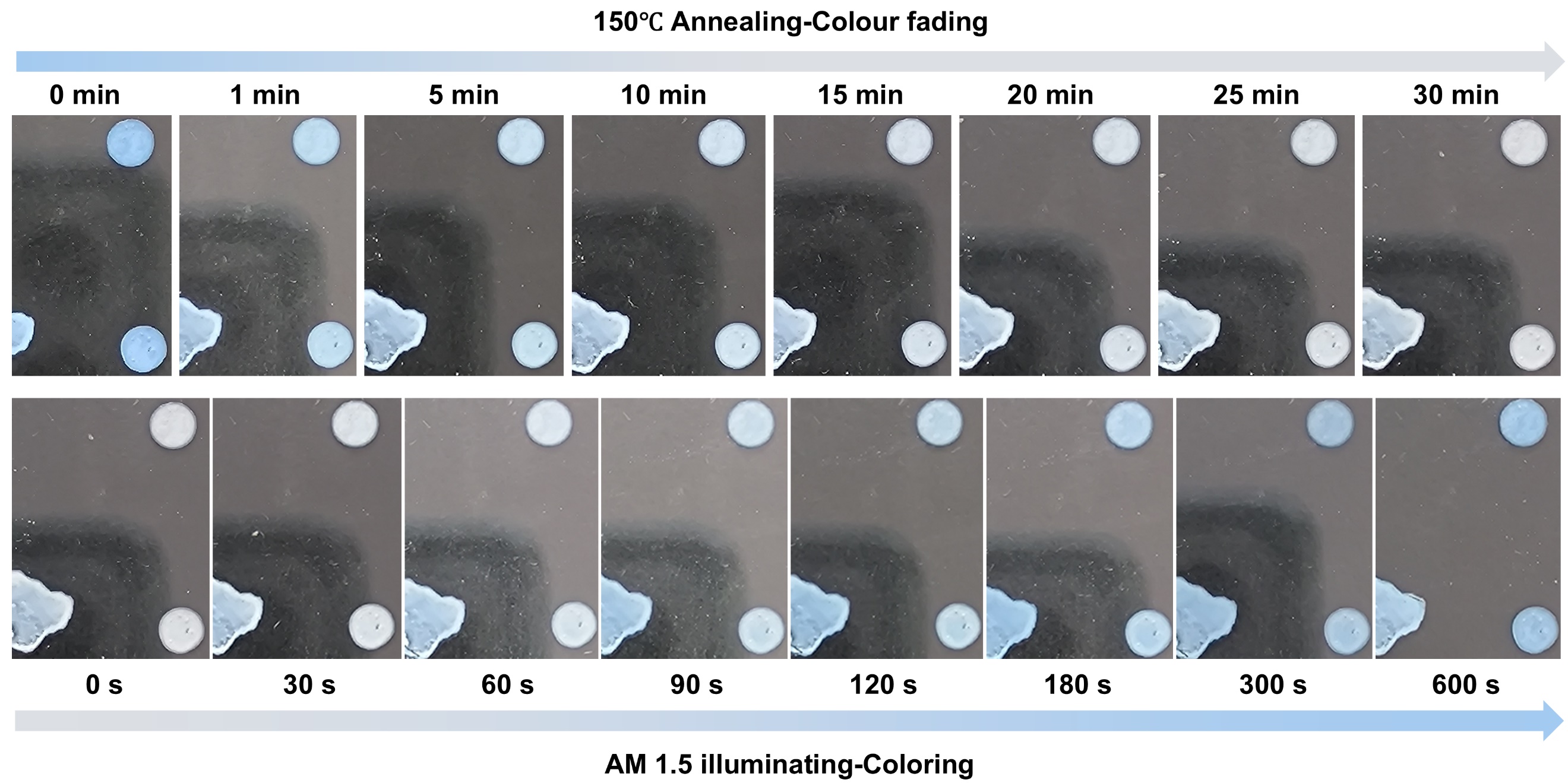


**Figure S1.** Color changes of Zn/WO_X_ (Zn/W=0.05) after annealing at 150 °C for 30 minutes followed by 10 minutes of irradiation under AM 1.5 light source. The observed changes are consistent with the typical bleaching-coloration cycle phenomenon of W_18_O_49_ materials.


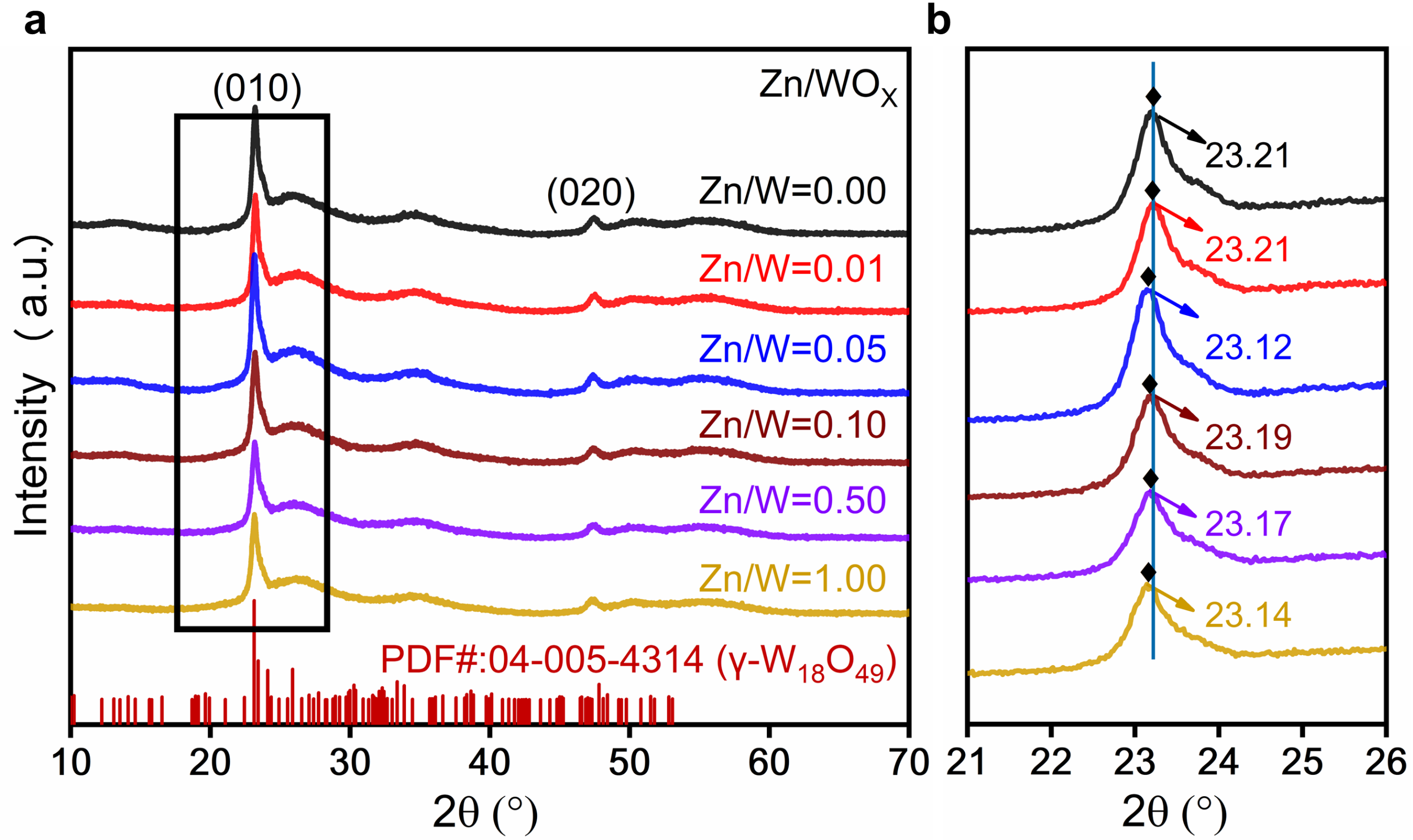


**Figure S2.** (a) XRD patterns of Zn/WO_X_ with different Zn^2+^ doping concentrations and (b) enlarged views of the (010) crystal plane.


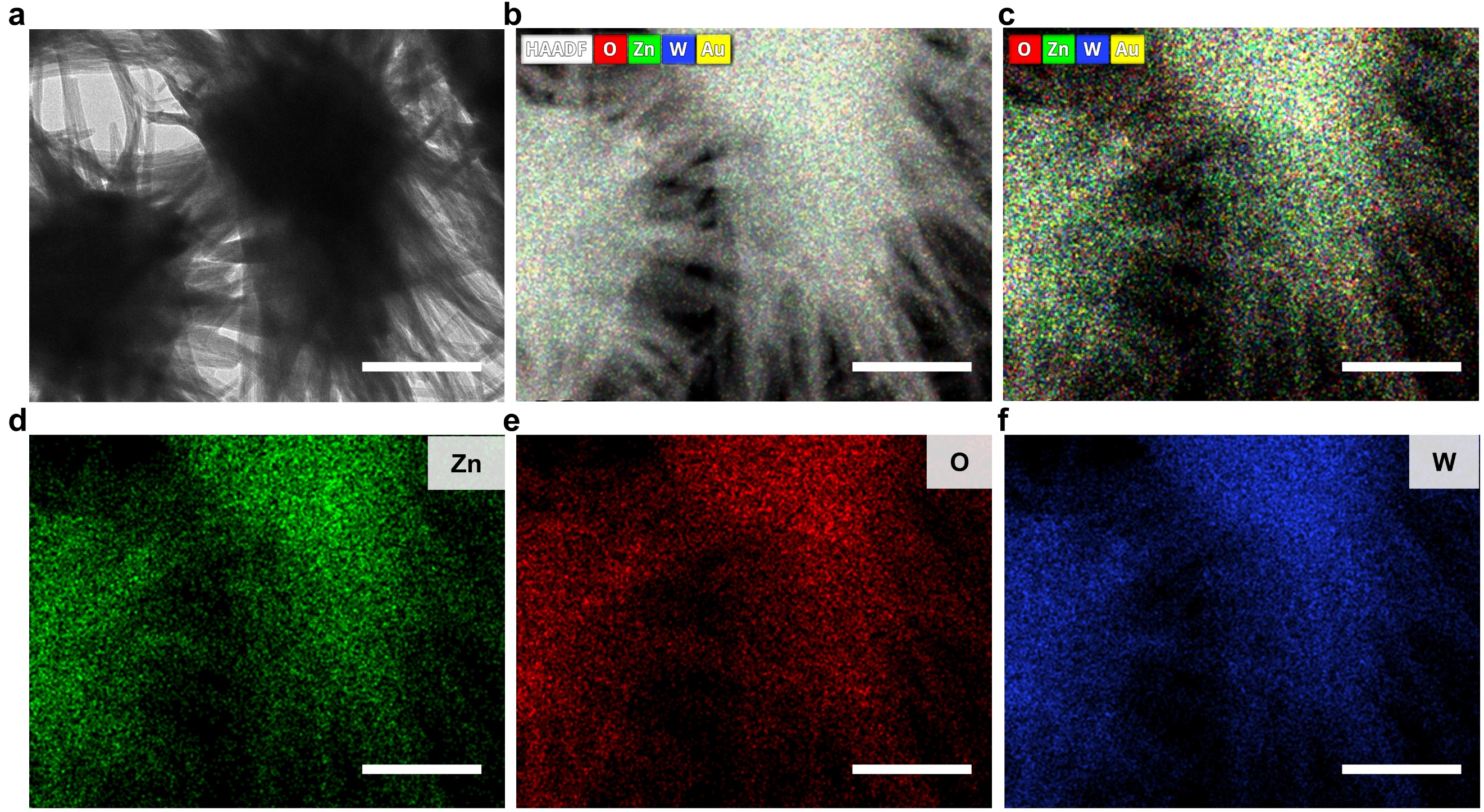


**Figure S3.** (a) TEM image and (b-f) EDS mapping of the Zn/WO_X_ (Zn/W=0.05) sample. Scale bar: 200 nm


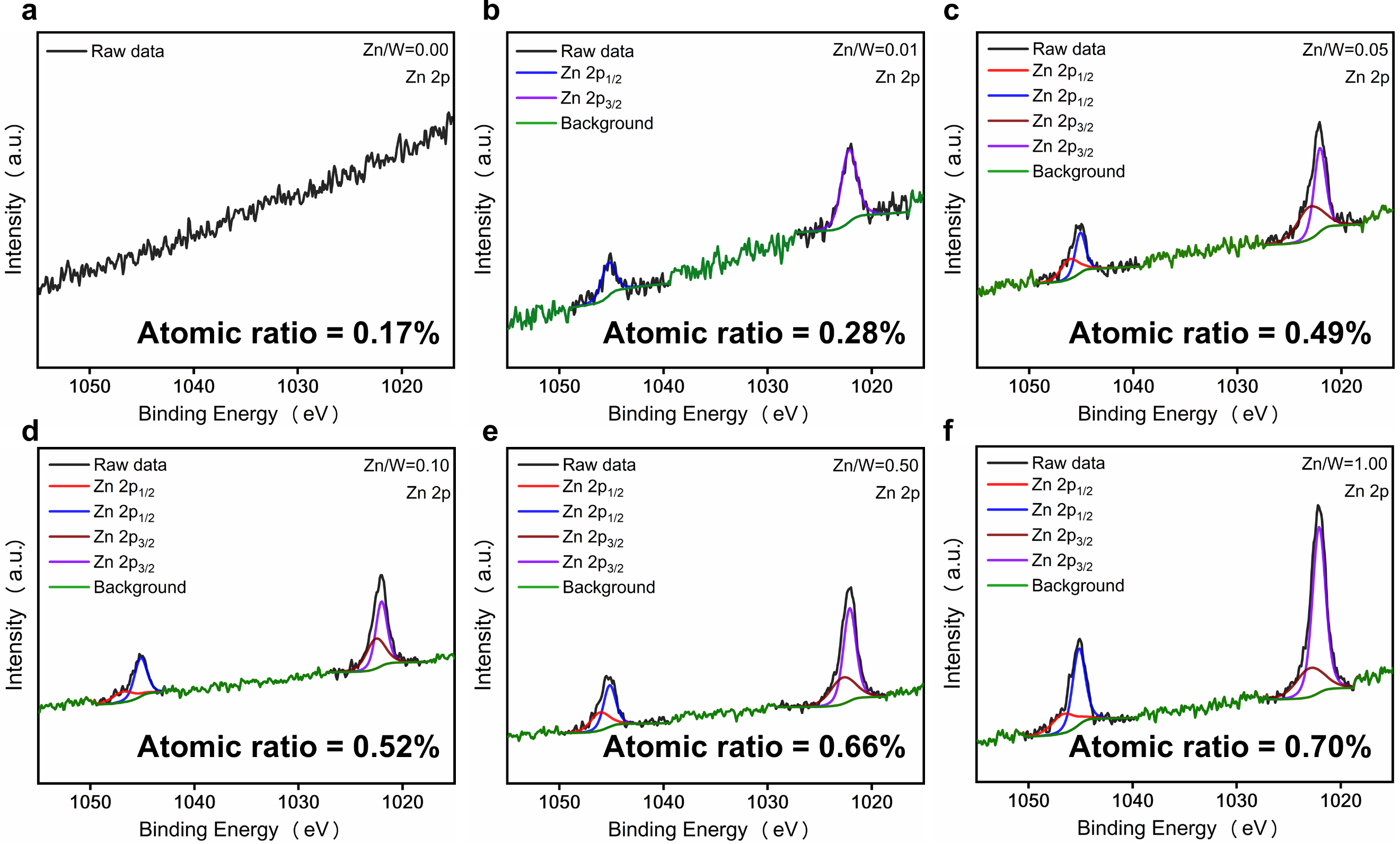


**Figure S4.** Zn 2p XPS fine spectra of Zn/WO_X_ with different Zn^2+^ doping concentrations. The atomic ratios indicated in the figure represent the actual incorporated Zn atomic percentages. (a) Zn/W=0.00, (b) Zn/W=0.01, (c) Zn/W=0.05, (d) Zn/W=0.10, (e) Zn/W=0.50, (f) Zn/W=1.00.


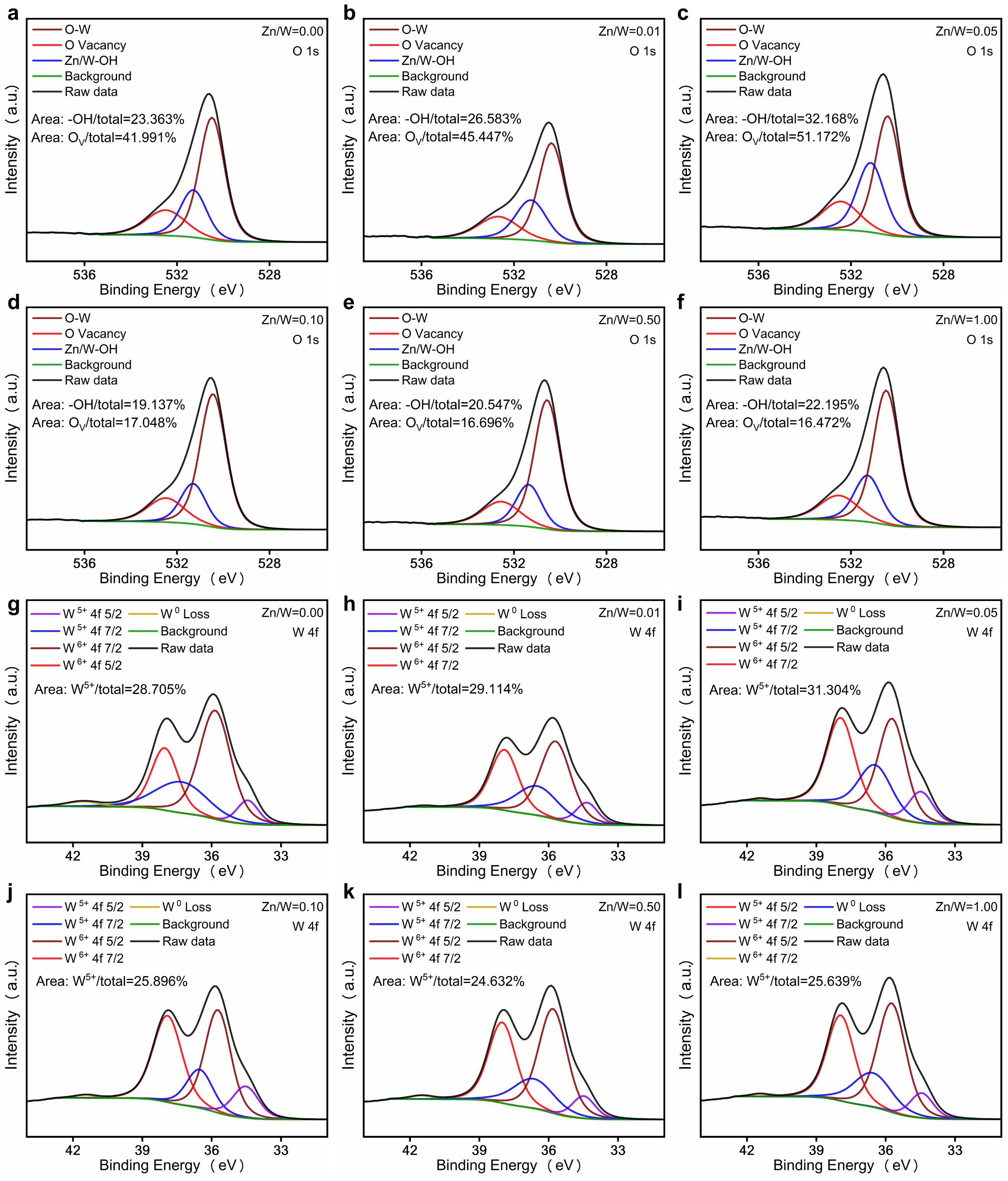


**Figure S5.** (a-f) O 1s XPS fine spectra of Zn/WO_X_ with different Zn^2+^ doping concentrations. The area ratios of the -OH peak and oxygen vacancy peak to the total peak are labeled in the figure to illustrate the changes in surface -OH group and oxygen vacancy content. (a) Zn/W=0.00, (b) Zn/W=0.01, (c) Zn/W=0.05, (d) Zn/W=0.10, (e) Zn/W=0.50, (f) Zn/W=1.00. (g-l) W 4f XPS fine spectra of Zn/WO_X_ with different Zn^2+^ doping concentrations. The area ratios of the W^5+^ peak to the total peak are labeled in the figure to illustrate the changes in W^5+^ content. (g) Zn/W=0.00, (h) Zn/W=0.01, (i) Zn/W=0.05, (j) Zn/W=0.10, (k) Zn/W=0.50, (l) Zn/W=1.00.





**Figure S6.** FTIR spectra of Zn/WO_X_ with different Zn^2+^ doping concentrations.


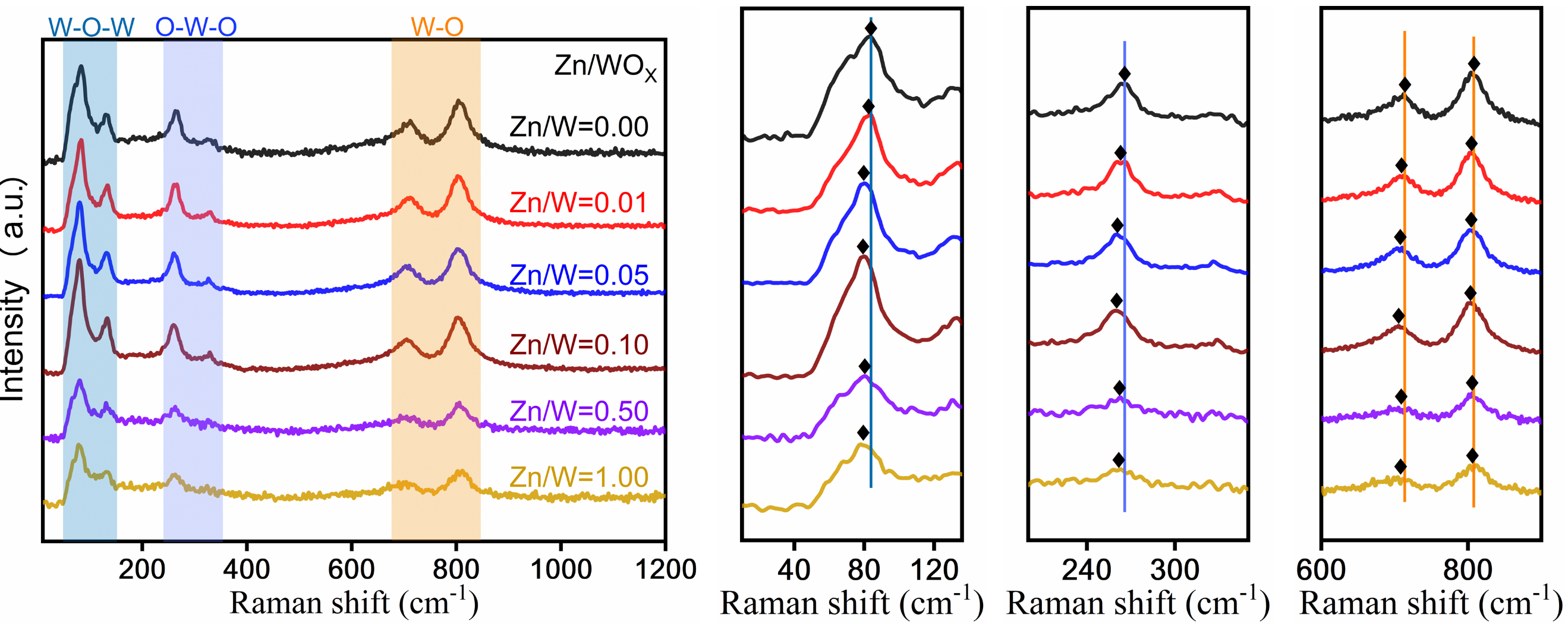


**Figure S7.** Raman spectra of Zn/WO_X_ with different Zn^2+^ doping concentrations.


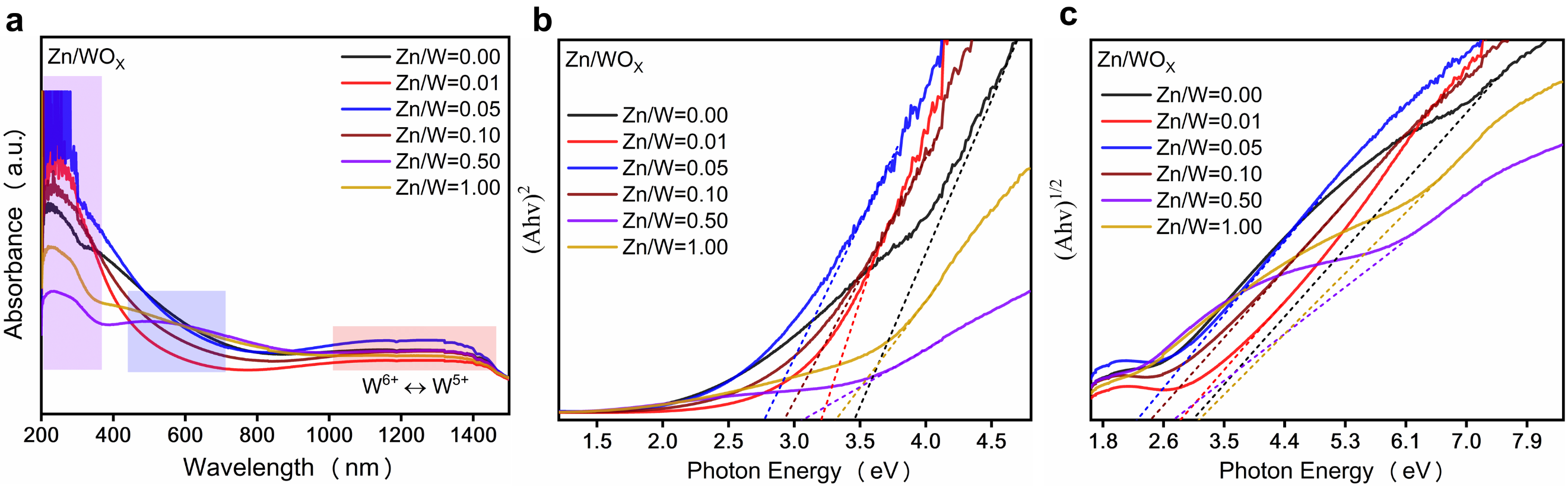


**Figure S8.** (a) UV-Vis absorption spectra and (b) direct bandgap Tauc plots, (c) indirect bandgap Tauc plots of Zn/WO_X_ with different Zn^2+^ doping concentrations.


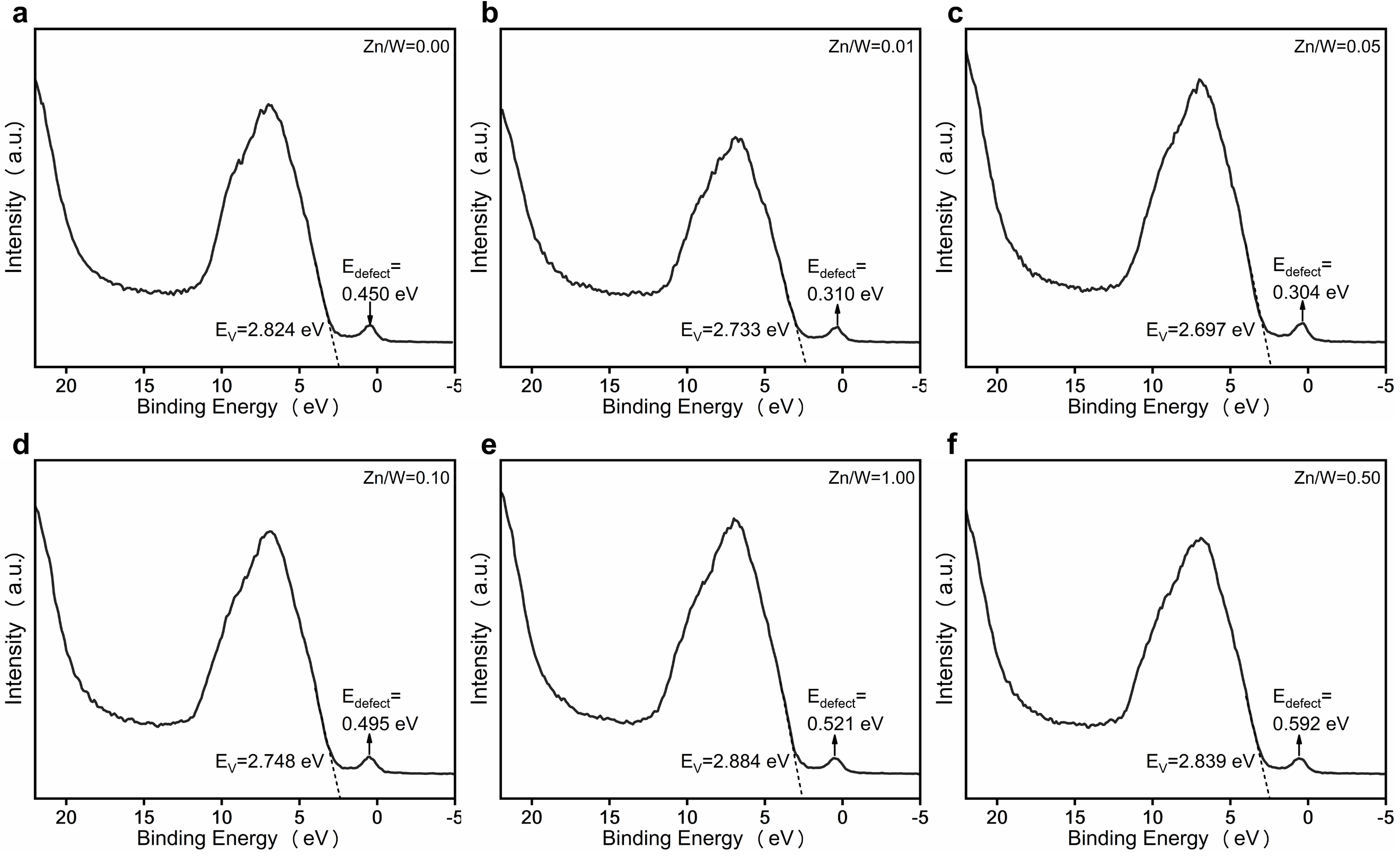


**Figure S9.** XPS valence band spectra of Zn/WO_X_ with different Zn^2+^ doping concentrations. (a) Zn/W=0.00, (b) Zn/W=0.01, (c) Zn/W=0.05, (d) Zn/W=0.10, (e) Zn/W=0.50, (f) Zn/W=1.00.


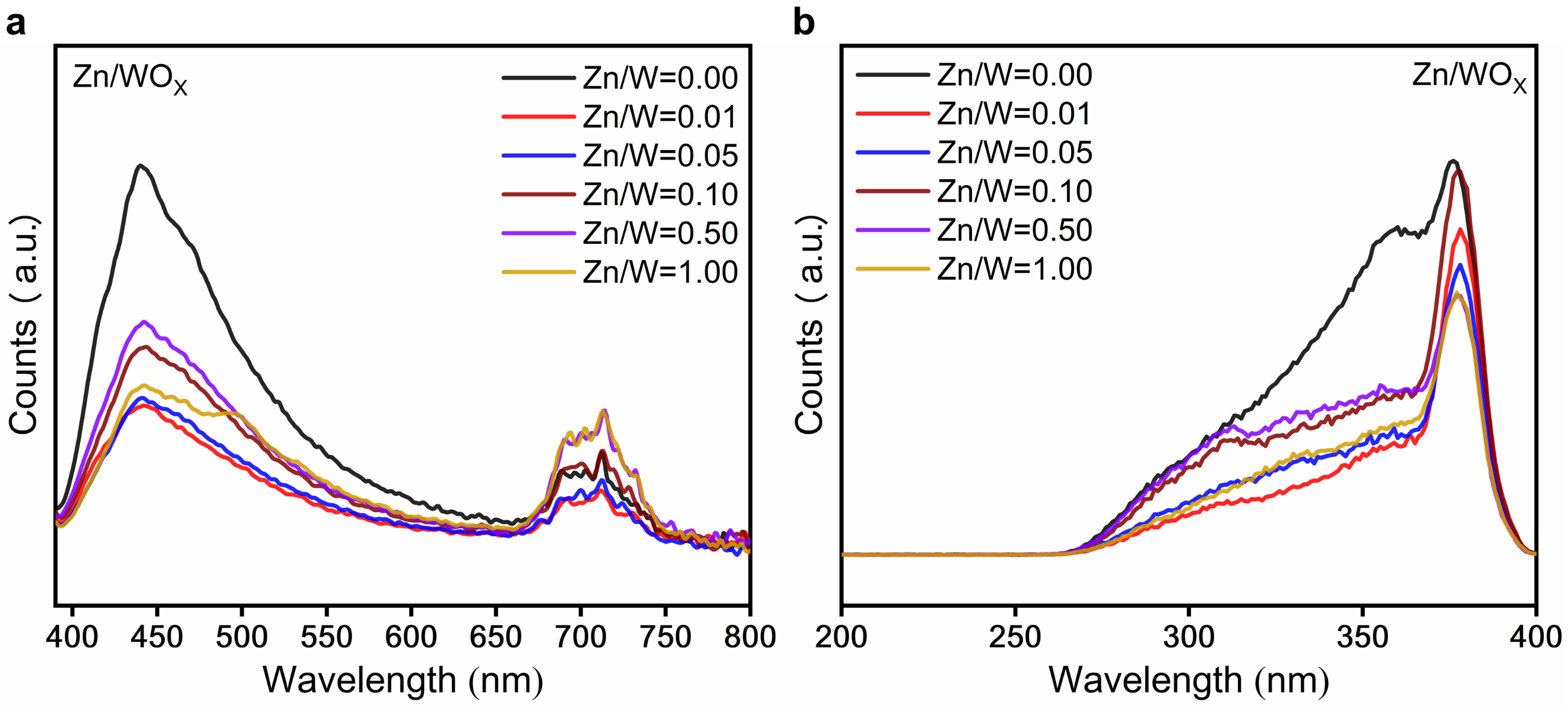


**Figure S10.** The (a) emission spectra and (b) excitation spectra of photoluminescence spectroscopy of Zn/WO_X_ with different Zn^2+^ doping concentrations.


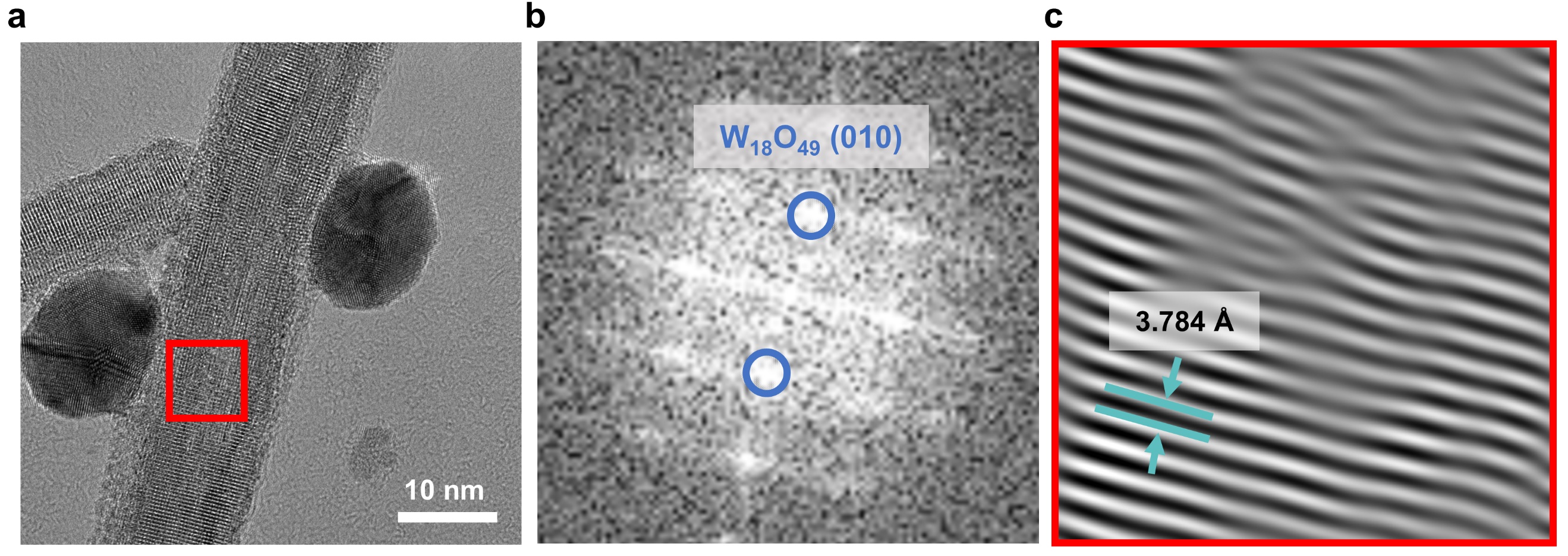


**Figure S11.** (a) TEM image, (b) SAED and (c) lattice fringe image of Zn/WO_X_.

**
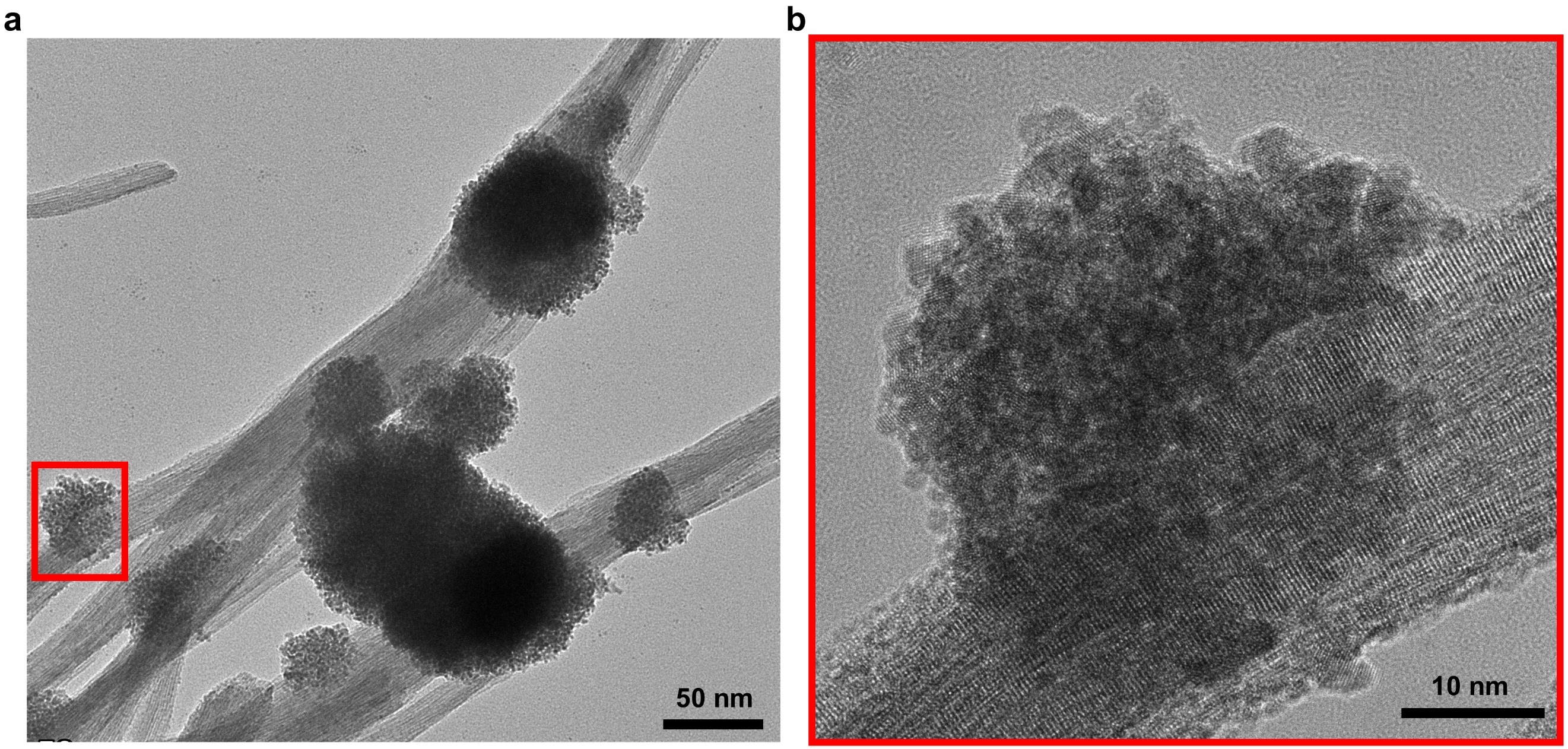
**

**Figure S12.** (a) TEM image of Zn/WOX@Au@Pt, (b) is an enlarged image of Pt nanoclusters.

**
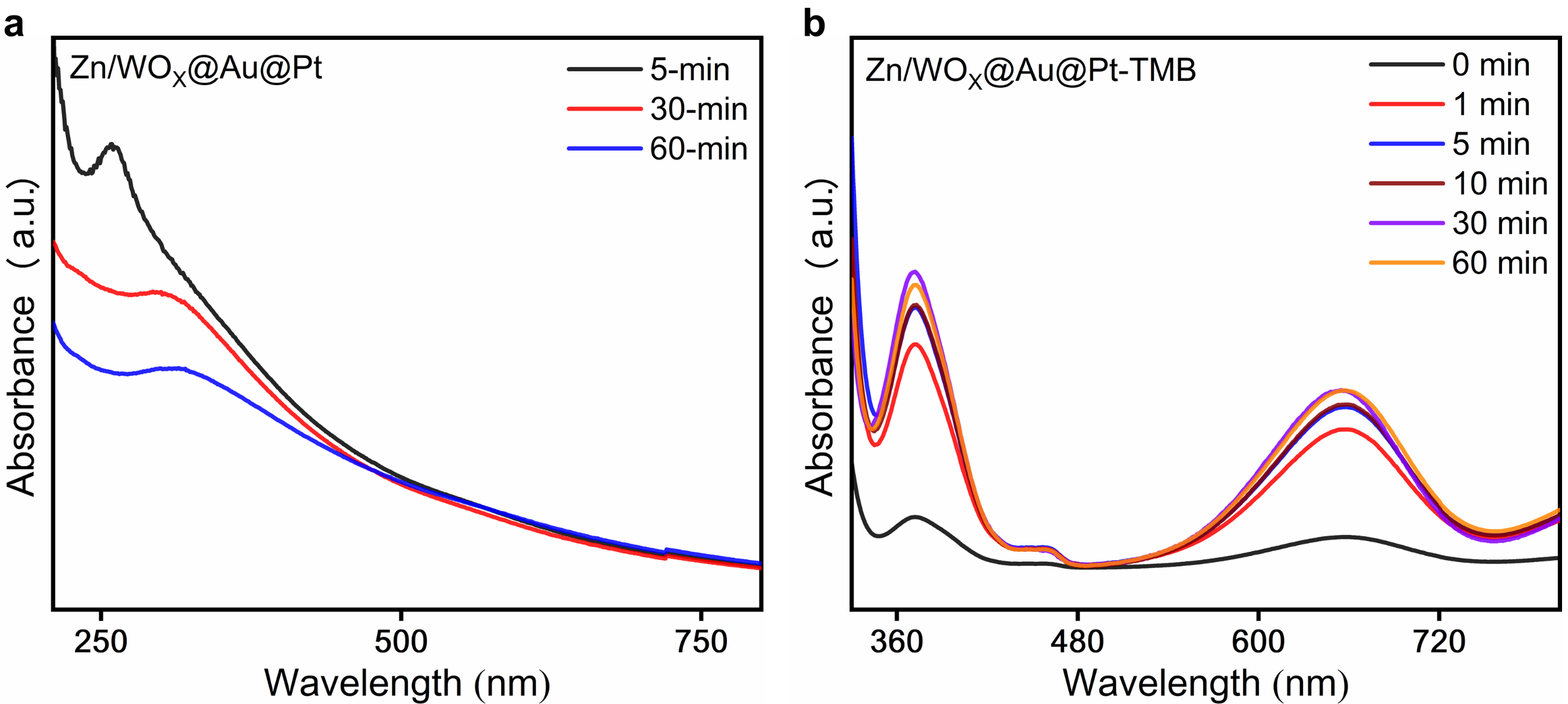
**

**Figure S13.** (a) UV-Vis absorption spectra of Zn/WO_X_@Au after different durations of light-induced reductive deposition of Pt; (b) UV-Vis absorption spectra of the TMB oxidation reaction catalyzed by Zn/WO_X_@Au@Pt (obtained after 5-minute light induction) at different time intervals.

**
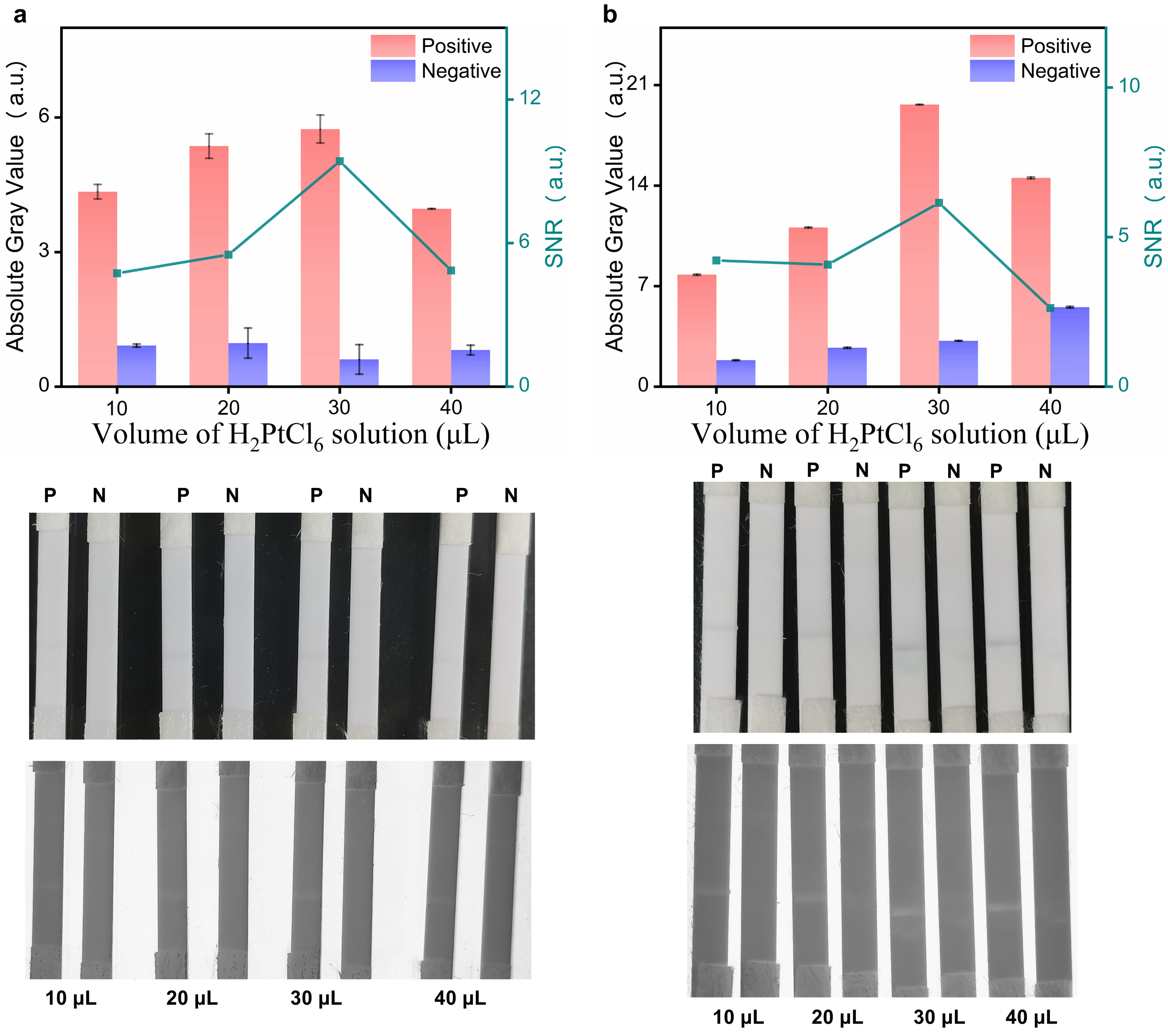
**

**Figure S14.** (a) Comparison of signal-to-noise ratios (SNR) of grayscale values on T obtained with different chloroplatinic acid addition amounts under ECM; (b) Comparison of signal-to-noise ratios of grayscale values on T obtained with different chloroplatinic acid addition amounts under CECM. The corresponding photos and grayscale values of test strips are shown in the lower part of the picture.

**
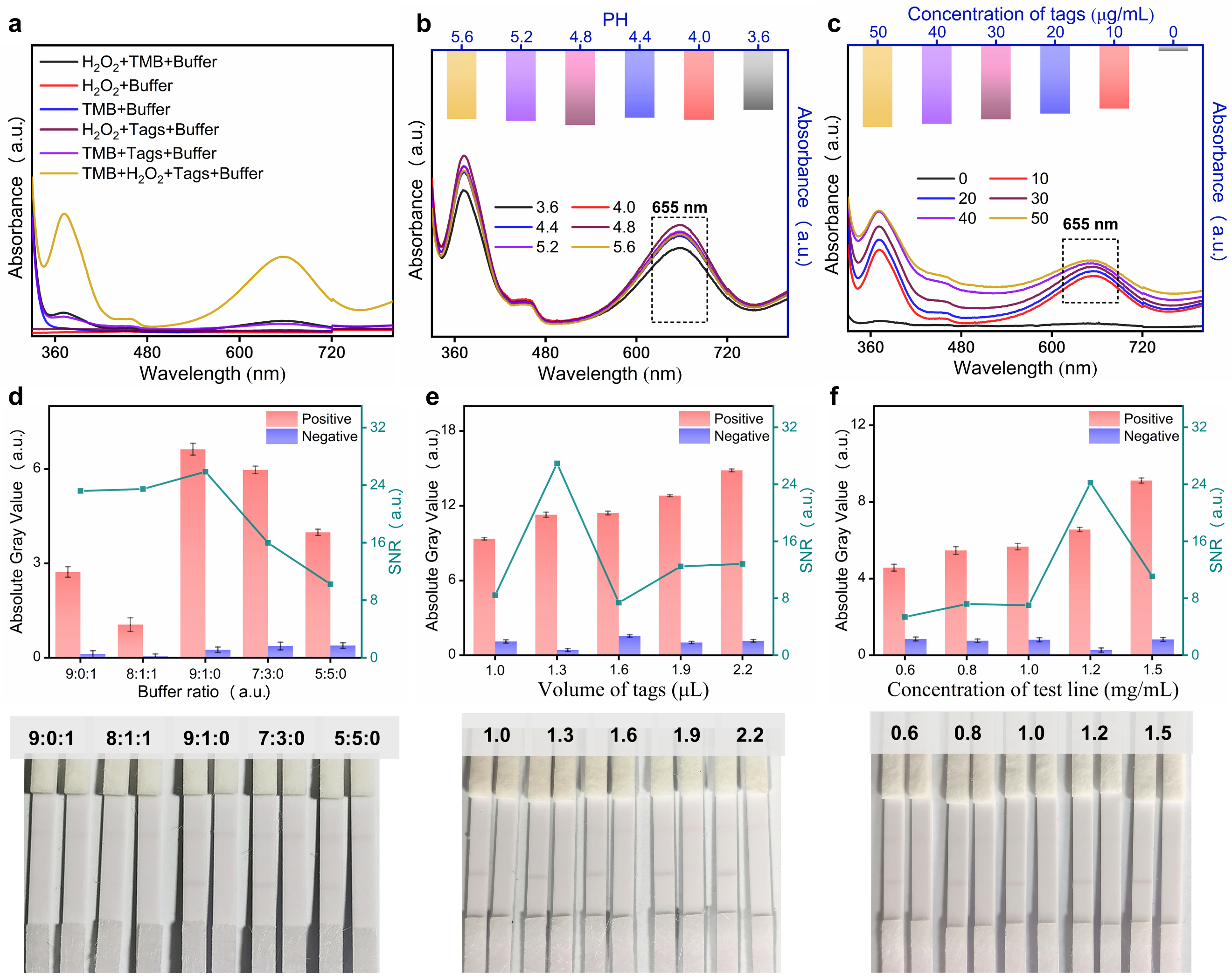
**

**Figure S15.** (a) UV-Vis absorption spectra of the color-developing solution composed of H_2_O_2_ + TMB + tags + NaAc-HAc buffer and the control groups; (b) UV-vis absorption spectra of the color-developing solutions prepared with NaAc-HAc buffers of different pH values; (c) UV-vis absorption spectra of the color-developing solutions prepared with different concentrations of Zn/WO_X_@Au@Pt tags; Under CECM, (d) signal-to-noise ratio (SNR) under different Buffer ratios (1% PBST : 10% BSA : FBS), (e) signal-to-noise ratio (SNR) under different volume of tags, (f) signal-to-noise ratio (SNR) under different antibody concentrations on the test line. The values used for calculating the SNR are all derived from the gray-scale values of the T area on the test strip.

**
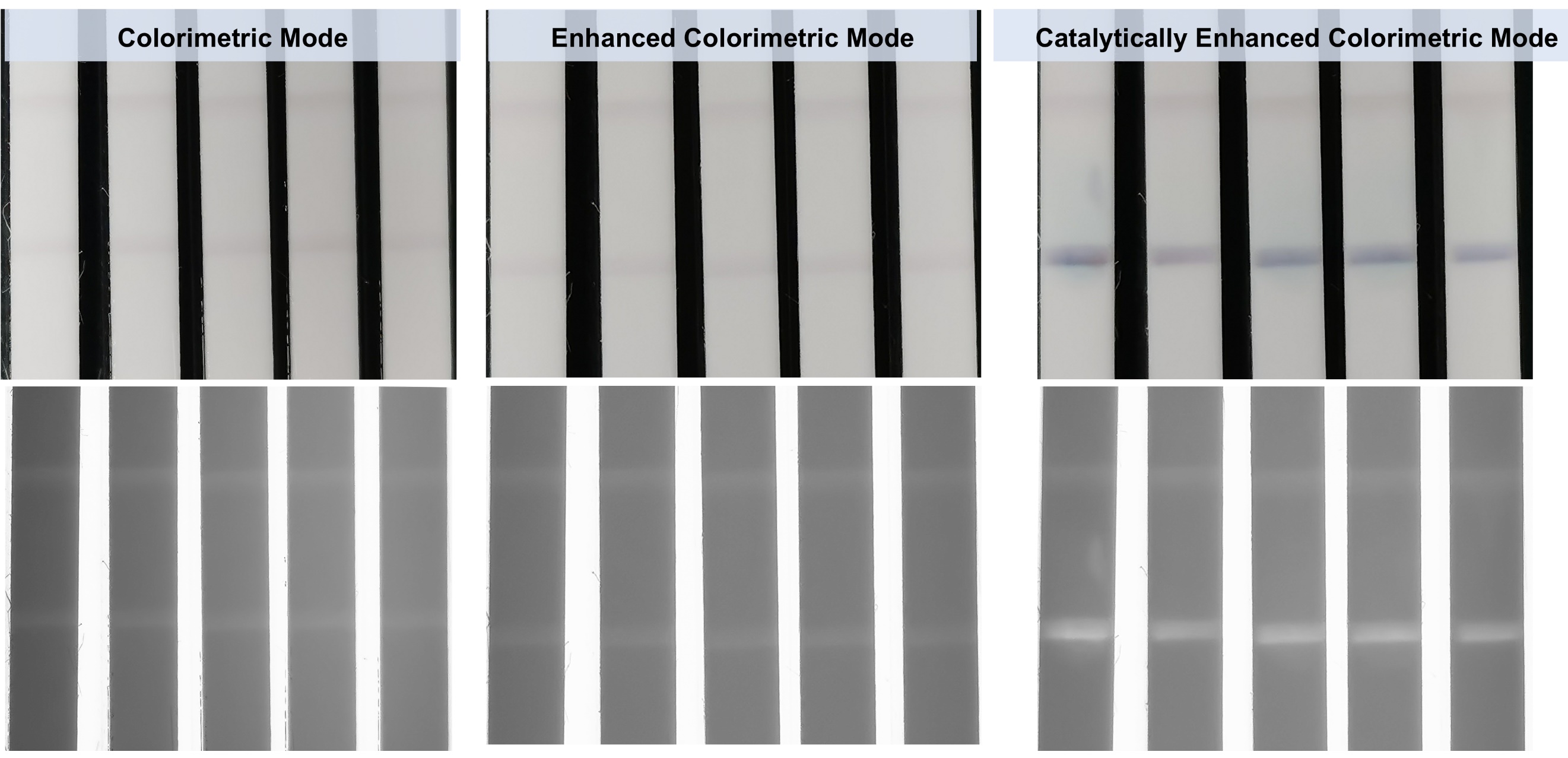
**

**Figure S16.** Five replicate tests were performed for 50 ng/mL Tcd B under CM, ECM, and CECM modes, respectively. The figure shows the corresponding test strip images and grayscale value images of the results for each mode.


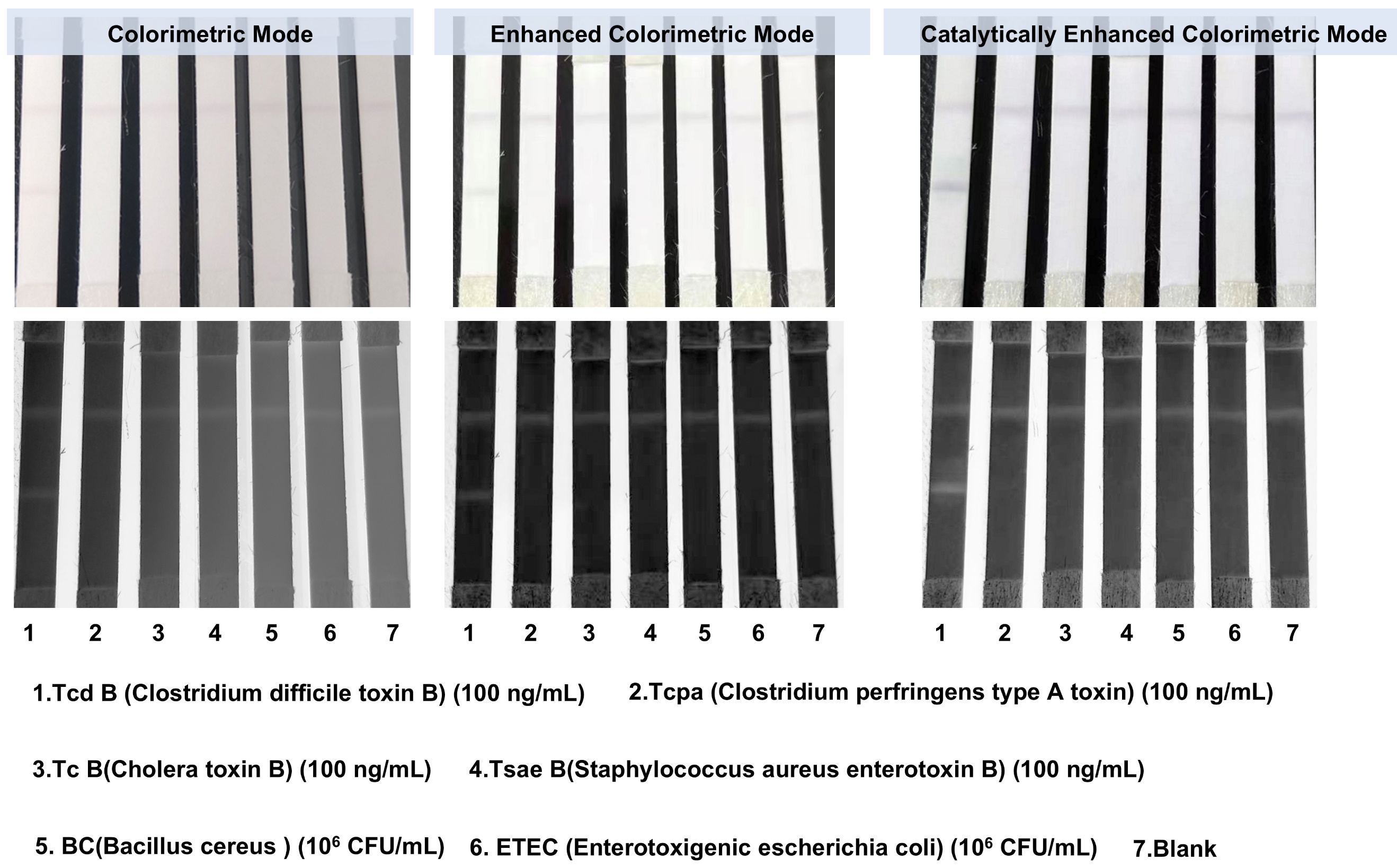


**Figure S17.** Tests were performed for six enterotoxin-related antigens and a blank control under CM, ECM, and CECM modes, respectively. The figure displays the test strip images and grayscale value images of the results for each mode. Note: The concentration of each toxin was 100 ng/mL, and the concentration of each bacterial solution was 10^6^ CFU/mL.

**Table S1.** Specific activity of Zn/WO_X_, Zn/WO_X_@Au and Zn/WO_X_@Au@Pt nanozymes under the conditions of 2 mM TMB, 200 mM H_2_O_2_, 200 μL reaction volume, pH=4.8 and room temperature.

| **Sample** | Zn/WO_X_@Au@Pt  (Zn/W=0.00) | Zn/WO_X_@Au@Pt  (Zn/W=0.05) | Zn/WO_X_@Au@Pt  (Zn/W=0.01) | Zn/WO_X_@Au  (Zn/W=0.05) | Zn/WO_X_  (Zn/W=0.05) |
| --- | --- | --- | --- | --- | --- |
| **specific activity based on Zn/WO_X_**  **(U/mg)** | 5.65 | 9.65 | 3.99 | 7.92 | 6.53 |
| **specific activity based on Au**  **(U/mg)** | 288.21 | 492.54 | 203.72 | 404.43 | ∞ |
| **specific activity based on Pt**  **(U/mg)** | 319.15 | 545.42 | 225.59 | ∞ | ∞ |

***Supplementary references***

[1]. Kresse, G.; Furthmuller, J.Efficient Iterative Schemes for Ab Initio Total-Energy calculations

Using a Plane-Wave Basis Set. *Phys. Rev. B.* **1996**, 54, 11169-11186.

[2]. Perdew, J. P., Burke, K. & Ernzerhof, M. Generalized gradient approximation made simple. *Phys. Rev. Lett*. **1996**, 77, 3865–3868.

[3]. Kresse, G.; Joubert, J. From Ultrasoft Pseudopotentials to the Projector Augmented-Wave

Method. *Phys. Rev. B*. **1999**, 59, 1758-1775.

[4]. Grimme, S.; Antony, J.; Ehrlich, S.; Krieg, H. A consistent and accurate ab initio parametrization of density functional dispersion correction (DFT-D) for the 94 elements H-Pu. *J. Chem. Phys.* **2010**, *132*.

[5]. Grimme, S.; Ehrlich, S.; Goerigk, L. Effect of the damping function in dispersion corrected density functional theory. *J Comput Chem* **2011**, *32*, 1456-1465.

[6]. Monkhorst, H. J.; Pack, J. D., Special points for Brillouin-zone integrations. *Phys. Rev. B.* **1976**, 13, 5188-5192.
